# Supplementary material for: A comparative multi-method re-analysis of the longitudinal evidence for causal intergroup contact effects on attitudes
Source: Commun Psychol. 2026 Jul 15;4:107. doi: 10.1038/s44271-026-00495-8 (PMC13372666; doi:10.1038/s44271-026-00495-8)
Supplement: Supplementary file 2 — Supplementary information [file 44271_2026_495_MOESM2_ESM.pdf]

Supplementary Table 1: Overview and Explanation of the Deviations between the Pre-Registration and the Final Manuscript

**Quote of the preregistration**	**Quote of the final manuscript and *highlight* of the differences**	**Explanation**
"Dependent variables include any operationalization of outgroup attitudes (e.g., feeling thermometer ratings or other individual-level cognitive, affective and behavioural (-intentional) measures)."	"assess any form of outgroup attitudes or a related construct (i.e., individual-level cognitive, affective and behavioural [-intentional] measures related to an outgroup) as dependent variable *under the condition that theoretically-based predictions about the construct's relation with intergroup contact could be derived*"	We added the qualifier that the attitudes variable should allow for theory-based predictions of the relation with intergroup contact. Otherwise, it would have been very difficult to identify whether a found effect would be confirming theoretical expectations. Some of the reviewed studies included attitude variables for which it was not possible to make such predictions.
"In this re-analysis, we will include the data from research articles on the longitudinal relation between intergroup contact and outgroup attitudes published in English in peer-reviewed journals until June 30th, 2020."	"be published before the *beginning of October 2022* in peer-reviewed journals in English language"	Given that this project suffered from substantial delays caused by the COVID-19 pandemic, we concluded the re-analysis much later than expected, and therefore decided to use a later reference date for the data inclusion.
"In case of convergence problems, we will try to simplify the models by using manifest instead of latent variables or by reducing the number of dependent variables in the model. When available, we will include stable and time-variant control variables."	"We used *observed* single- or multi-item measures for contact and attitudes (…)."	We decided to model all reanalysis based on observed/manifest variables instead of latent factor scores because the complexity of the models was very high (leading to convergence problems if latent variable modelling was applied) and because most original publications used single-item measures or observed mean or sum scores.
"When available, we will include stable and time-variant control variables."	/	We did not include control variables in our analysis as the re-analysed datasets varied strongly in the number and nature of potential control variables.

We did not preregister the testing for stationarity, the model-fit comparison between the CLPM and the RI-CLPM and the internal meta-analysis to summarise our results.

Supplementary Table 2: Model Fit Results of the (Partial) Metric Measurement Invariance Testing

Dataset and Model information			Model fit						Testing for measurement invariance			
Dataset	Construct	Model	χ^2-value	df	*p*	CFI	RMSEA	SRMR	MLR-corrected χ^2-difference value	df	*p*	Preferred model
Bohrer et al. (2019)	Positive contact	Configural	6.143	13	.941	1.000	.000	.012	0.488	3	.922	Metric
		Metric	6.839	16	.976	1.000	.000	.013				
	Evaluation	Configural	6.084	13	.943	1.000	.000	.007	2.302	3	.512	Metric
		Metric	8.311	16	.939	1.000	.000	.015				
Hässler et al. (2019)	Outgroup trust	Configural	6.589	5	.253	.998	.031	.018	0.799	2	.671	Metric
		Metric	7.481	7	.381	.999	.013	.021				
Jugert et al. (2011)	Affective attitudes	Configural*	104.650	45	<.001	.930	.117	.060				
		Metric*	110.737	51	<.001	.930	.109	.064	7.402	6	.285	Metric
	Outgroup orientation	Configural*	59.619	45	.071	.985	.051	.056				
		Metric*	69.347	51	.045	.981	.052	.070	9.920	6	.128	Metric
Kotzur & Wagner (2021)	Positive contact	Configural	4.116	5	.533	1.000	.000	.014				
		Metric	10.612	7	.156	.997	.031	.029	6.795	2	.033	Configural
	Negative contact	Configural	8.556	5	.128	.996	.043	.023				
		Metric	11.142	7	.133	.996	.037	.029	2.418	2	.299	Metric
	Prejudice	Configural	4.304	5	.506	1.000	.000	.006				
		Metric	6.080	7	.530	1.000	.000	.010	1.785	2	.410	Metric
Swart et al. (2011)	Cross-group friendships	Configural	4.710	5	.452	1.000	.000	.011				
		Metric	5.487	7	.601	1.000	.000	.015	0.687	2	.709	Metric
	Outgroup attitudes	Configural*	143.285	45	<.001	.892	.079	.066				
		Partial metric*	150.343	49	<.001	.886	.078	.068	8.150	4	.086	Partial metric
	Negative action tendencies	Configural*	19.499	21	.553	1.000	.000	.024				
		Metric*	23.459	25	.551	1.000	.000	.032	3.957	4	.412	Metric

Swart et al. (2011)	Outgroup variability	Configural	15.206	5	.010	.974	.072	.027				
		Metric	15.435	7	.031	.969	.061	.031	1.317	2	.518	Metric
Vezzali et al. (2022) – Immigrant sample	Contact quantity	Configural*	42.790	13	<.001	.963	.112	.055				
		Metric	45.936	16	<.001	.964	.100	.055	2.207	3	.531	Metric
	Contact quality	Configural	137.660	92	.001	.957	.058	.061				
		Metric	145.887	101	.002	.956	.056	.074	9.182	9	.421	Metric
	Explicit positive attitudes	Configural	185.432	92	<.001	.866	.080	.073				
		Metric	195.671	101	<.001	.857	.078	.083	11.754	9	.228	Metric
	Explicit negative attitudes	Configural	176.241	92	<.001	.876	.074	.069				
		Metric	191.798	101	<.001	.861	.075	.075	15.919	9	.069	Metric
Vezzali et al. (2022) – Italian sample	Contact quantity	Configural	38.729	13	<.001	.977	.076	.036				
		Metric	41.190	16	.001	.977	.067	.036	1.907	3	.592	Metric
	Contact quality	Configural	195.017	92	<.001	.965	.057	.041				
		Partial metric	202.581	98	<.001	.963	.057	.048	8.460	6	.206	Partial metric
	Explicit positive attitudes	Configural	218.675	92	<.001	.938	.066	.049				
		Partial metric	228.736	98	<.001	.936	.065	.052	9.647	6	.140	Partial metric
	Prejudice	Configural	186.716	92	<.001	.932	.056	.053				
		Metric	196.730	101	<.001	.930	.054	.064	11.194	9	.263	Metric
Wagner et al. (2008)	Positive contact	Configural	36.748	21	.018	.995	.033	.017				
		Metric	40.983	25	.023	.995	.030	.022	3.582	4	.466	Metric
	Prejudice	Configural	0.748	5	.980	1.000	.000	.002				
		Metric	0.955	7	.996	1.000	.000	.004	0.191	2	.909	Metric

Notes. We specified a construct factor per measurement time including at least two construct indicators and at least three measurement times. Additionally, for each construct indicator but the first one (which scales the latent variable, i.e., its unstandardized factor loading is set to 1), we defined indicator-specific factors with the identical construct indicators from all measurement times loading on these factors with unstandardized factor loadings set to 1. The indicator-specific factors did not correlate with the substantive construct factors. Df = degrees of freedom, p = p-value, CFI =

Comparative Fit Index, RMSEA = Root Mean Square Error of Approximation, SRMR = Standardized Root Mean Square Residual. * The covariance matrix was not positive definite, resulting in implausible parameter estimates and less trustworthy model results.

Supplementary Table 3: Model Fit Information for Freely Estimated and Stationarity CLPM and RI-CLPM Models

Dataset and Model information				Model fit						Testing for stationarity			
IVs	DVs	Model	Station-arity	χ² -value	df	*p*	CFI	RMSEA	SRMR	MLR-corrected χ²-difference value	df	*p*	Preferred model
Bagci et al. (2022)													
Positive contact	Avoidance tendencies	CLPM	No	40.513	4	<.001	.891	.145	.055	3.681	4	.451	Station-arity
			Yes	47.111	8	<.001	.893	.101	.056				
		RI-CLPM	No	0.028	1	.868	1.000	.000	.001	9.164	4	.057	Stationari-ty
			Yes	9.500	5	.091	.990	.039	.026				
Negative contact	Avoidance tendencies	CLPM	No	21.234	4	<.001	.916	.100	.037	2.240	4	.692	Stationari-ty
			Yes	24.229	8	.002	.924	.067	.039				
		RI-CLPM	No	1.009	1	.315	1.000	.000	.009	0.668	4	.955	Stationari-ty
			Yes	1.638	5	.897	1.000	.000	.011				
Positive and negative contact	Avoidance tendencies	CLPM	No	43.258	9	<.001	.920	.092	.039	8.013	9	.533	Stationari-ty
			Yes	52.461	18	<.001	.924	.064	.043				
		RI-CLPM	No	3.659	3	.301	.999	.017	.013	9.936	9	.356	Stationari-ty
			Yes	13.563	12	.329	.998	.011	.025				
Positive contact	Approach tendencies	CLPM	No	24.773	4	<.001	.976	.109	.038	7.706	4	.103	Stationari-ty
			Yes	31.743	8	<.001	.972	.083	.048				
		RI-CLPM	No	0.502	1	.479	1.000	.000	.006	4.862	4	.302	Stationari-ty
			Yes	5.448	5	.364	1.000	.011	.022				
Negative contact	Approach tendencies	CLPM	No	22.149	4	<.001	.960	.099	.028	0.822	4	.935	Stationari-ty
			Yes	22.891	8	.004	.967	.063	.028				
		RI-CLPM	No	0.042	1	.837	1.000	.000	.002	1.521	4	.823	Stationari-ty
			Yes	1.582	5	.903	1.000	.000	.010				
Positive and	Approach	CLPM	No	31.176	9	<.001	.977	.073	.029	14.375	9	.109	Stationari-

negative contact	tendencies		Yes	45.176	18	<.001	.973	.057	.037				ty
		RI-CLPM	No	3.335	3	.343	1.000	.013	.010	8.677	9	.468	Stationarity
			Yes	11.975	12	.448	1.000	.000	.020				
Positive contact	Approach and avoidance tendencies	CLPM	No	44.541	9	<.001	.969	.094	.039	17.531	9	.041	Freely estimated
			Yes	62.815	18	<.001	.963	.073	.046				
		RI-CLPM	No	1.005	3	.800	1.000	.000	.006	12.425	9	.190	Stationarity
			Yes	13.674	12	.322	.999	.013	.021				
Negative contact	Approach and avoidance tendencies	CLPM	No	38.778	9	<.001	.959	.086	.032	4.633	9	.865	Stationarity
			Yes	45.043	18	<.001	.964	.057	.033				
		RI-CLPM	No	2.104	3	.551	1.000	.000	.008	3.735	9	.928	Stationarity
			Yes	5.791	12	.926	1.000	.000	.011				
Positive and negative contact	Approach and avoidance tendencies	CLPM	No	51.996	16	<.001	.972	.070	.031	22.402	16	.131	Stationarity
			Yes	75.040	32	<.001	.968	.053	.038				
		RI-CLPM	No	5.584	6	.471	1.000	.000	.010	15.100	16	.517	Stationarity
			Yes	20.688	22	.540	1.000	.000	.020				
Barlow et al. (2019) – Non-Asian sample													
Positive contact	Warmth	CLPM	No	1,996.634	12	<.001	.853	.189	.099	16.198	8	.040	Freely estimated
			Yes	2,037.979	20	<.001	.852	.146	.099				
		RI-CLPM	No	11.811	9	.224	1.000	.009	.007	23.531	8	.003	Freely estimated
			Yes	35.520	17	.005	.999	.015	.010				
Negative contact	Warmth	CLPM	No	2,021.718	12	<.001	.795	.188	.099	9.969	8	.267	Stationarity
			Yes	2,045.432	20	<.001	.795	.146	.099				
		RI-CLPM	No	19.153	9	.024	.999	.015	.007	16.981	8	.030	Freely estimated
			Yes	36.133	17	.004	.998	.015	.010				
Positive and negative contact	Warmth	CLPM	No	2,760.764	27	<.001	.844	.144	.083	26.343	18	.092	Stationarity
			Yes	2,788.090	45	<.001	.844	.112	.084				
		RI-CLPM	No	35.379	21	.026	.999	.012	.008	40.169	18	.002	Freely

			Yes	75.737	39	<.001	.998	.013	.011				estimated
Positive contact	Anger	CLPM	No	1,815.788	12	<.001	.804	.176	.094	11.313	8	.185	Stationarity
			Yes	1,865.938	20	<.001	.803	.137	.095				
		RI-CLPM	No	9.541	9	.389	1.000	.000	.008	14.652	8	.066	Stationarity
			Yes	24.214	17	.114	.999	.008	.011				
Negative contact	Anger	CLPM	No	1,555.584	12	<.001	.785	.162	.097	4.755	8	.783	Stationarity
			Yes	1,579.359	20	<.001	.785	.125	.097				
		RI-CLPM	No	19.646	9	.020	.999	.014	.007	18.293	8	.019	Freely estimated
			Yes	37.950	17	.002	.997	.015	.010				
Positive and negative contact	Anger	CLPM	No	2,548.034	27	<.001	.813	.137	.082	20.140	18	.325	Stationarity
			Yes	2,583.602	45	<.001	.812	.106	.083				
		RI-CLPM	No	33.741	21	.039	.999	.009	.008	40.800	18	.002	Freely estimated
			Yes	74.708	39	.001	.998	.012	.011				
Positive contact	Warmth and anger	CLPM	No	2,647.049	27	<.001	.868	.140	.086	23.475	18	.173	Stationarity
			Yes	2,684.693	45	<.001	.868	.109	.087				
		RI-CLPM	No	26.217	21	.198	1.000	.008	.007	31.972	18	.022	Freely estimated
			Yes	58.291	39	.024	.999	.011	.010				
Negative contact	Warmth and anger	CLPM	No	2,593.793	27	<.001	.844	.138	.088	19.095	18	.386	Stationarity
			Yes	2,617.351	45	<.001	.844	.107	.088				
		RI-CLPM	No	43.219	21	.003	.999	.014	.008	34.878	18	.010	Freely estimated
			Yes	78.064	39	<.001	.998	.014	.010				
Positive and negative	Warmth and anger	CLPM	No	3,323.703	48	<.001	.867	.117	.077	37.156	32	.244	Stationarity
			Yes	3,359.631	80	<.001	.866	.090	.077				

contact		RI-CLPM	No	61.053	38	.010	.999	.011	.008	58.324	32	.003	Freely estimated
			Yes	119.476	70	<.001	.998	.011	.011				
Barlow et al. (2019) – Non-European New Zealander sample													
Positive contact	Warmth	CLPM	No	312.727	12	<.001	.842	.175	.102	9.093	8	.335	Stationarity
			Yes	325.488	20	<.001	.841	.136	.101				
		RI-CLPM	No	14.038	9	.121	.999	.019	.015	4.654	8	.794	Stationarity
			Yes	18.328	17	.368	1.000	.000	.018				
Negative contact	Warmth	CLPM	No	431.636	12	<.001	.739	.190	.100	4.179	8	.841	Stationarity
			Yes	442.337	20	<.001	.742	.147	.101				
		RI-CLPM	No	4.616	9	.866	1.000	.000	.010	4.986	8	.759	Stationarity
			Yes	9.625	17	.919	1.000	.000	.014				
Positive and negative contact	Warmth	CLPM	No	523.013	27	<.001	.823	.142	.087	15.969	18	.595	Stationarity
			Yes	542.727	45	<.001	.823	.110	.087				
		RI-CLPM	No	19.445	21	.557	1.000	.000	.013	10.135	18	.927	Stationarity
			Yes	29.320	39	.870	1.000	.000	.016				
Positive contact	Anger	CLPM	No	319.322	12	<.001	.796	.165	.092	11.823	8	.159	Stationarity
			Yes	335.830	20	<.001	.795	.128	.092				
		RI-CLPM	No	14.009	9	.122	.996	.028	.016	7.816	8	.452	Stationarity
			Yes	21.695	17	.197	.997	.016	.018				
Negative contact	Anger	CLPM	No	381.662	12	<.001	.737	.173	.105	9.295	8	.318	Stationarity
			Yes	401.839	20	<.001	.735	.135	.104				
		RI-CLPM	No	17.161	9	.046	.994	.031	.018	11.525	8	.174	Stationarity
			Yes	28.633	17	.038	.994	.023	.024				

Positive and negative contact	Anger	CLPM	No	519.093	27	<.001	.797	.136	.085	17.941	18	.460	Stationarity
			Yes	543.465	45	<.001	.797	.105	.085				
		RI-CLPM	No	27.463	21	.156	.997	.018	.016	14.713	18	.682	Stationarity
			Yes	41.884	39	.347	1.000	.000	.019				
Positive contact	Warmth and anger	CLPM	No	483.115	27	<.001	.846	.135	.090	20.603	18	.300	Stationarity
			Yes	514.831	45	<.001	.846	.105	.090				
		RI-CLPM	No	31.211	21	.070	.997	.023	.017	14.812	18	.675	Stationarity
			Yes	45.851	39	.209	.999	.011	.019				
Negative contact	Warmth and anger	CLPM	No	592.929	27	<.001	.790	.144	.093	15.847	18	.603	Stationarity
			Yes	621.677	45	<.001	.791	.111	.094				
		RI-CLPM	No	26.057	21	.204	.998	.017	.016	19.665	18	.352	Stationarity
			Yes	45.704	39	.214	.998	.010	.021				
Positive and negative contact	Warmth and anger	CLPM	No	690.449	48	<.001	.839	.117	.081	33.767	32	.382	Stationarity
			Yes	733.552	80	<.001	.839	.091	.082				
		RI-CLPM	No	46.880	38	.153	.998	.014	.016	28.125	32	.663	Stationarity
			Yes	74.764	70	.326	1.000	.000	.019				
Barlow et al. (2019) – Non-Māori sample													
Positive contact	Warmth	CLPM	No	1,743.377	12	<.001	.868	.183	.094	9.198	8	.326	Stationarity
			Yes	1,782.967	20	<.001	.868	.142	.094				
		RI-CLPM	No	16.006	9	.067	1.000	.013	.009	7.413	8	.493	Stationarity
			Yes	23.292	17	.140	.999	.010	.010				
Negative contact	Warmth	CLPM	No	1,844.506	12	<.001	.810	.189	.099	10.705	8	.219	Stationarity
			Yes	1,888.855	20	<.001	.810	.147	.099				

		RI-CLPM	No	21.237	9	.012	.999	.017	.010	11.678	8	.166	Stationarity
			Yes	32.752	17	.012	.998	.015	.012				
Positive and negative contact	Warmth	CLPM	No	2,508.288	27	<.001	.860	.143	.081	20.396	18	.311	Stationarity
			Yes	2,541.014	45	<.001	.860	.111	.081				
		RI-CLPM	No	36.269	21	.020	.999	.012	.009	22.229	18	.222	Stationarity
			Yes	58.261	39	.024	.999	.011	.012				
Positive contact	Anger	CLPM	No	1,708.203	12	<.001	.835	.173	.091	26.099	8	.001	Freely estimated
			Yes	1,750.788	20	<.001	.834	.135	.093				
		RI-CLPM	No	9.585	9	.385	1.000	.003	.006	20.894	8	.007	Freely estimated
			Yes	30.950	17	.020	.999	.011	.012				
Negative contact	Anger	CLPM	No	1,475.516	12	<.001	.822	.166	.099	27.170	8	<.001	Freely estimated
			Yes	1,524.736	20	<.001	.821	.129	.100				
		RI-CLPM	No	20.225	9	.017	.999	.015	.007	38.025	8	<.001	Freely estimated
			Yes	58.798	17	<.001	.996	.022	.017				
Positive and negative contact	Anger	CLPM	No	2,392.833	27	<.001	.844	.136	.082	37.828	18	.004	Freely estimated
			Yes	2,435.089	45	<.001	.843	.106	.083				
		RI-CLPM	No	34.786	21	.030	.999	.011	.007	46.014	18	<.001	Freely estimated
			Yes	81.325	39	<.001	.997	.015	.015				
Positive contact	Warmth and anger	CLPM	No	2,394.234	27	<.001	.883	.138	.085	36.562	18	.006	Freely estimated
			Yes	2,450.596	45	<.001	.883	.107	.085				
		RI-CLPM	No	31.672	21	.063	1.000	.010	.008	30.907	18	.030	Freely estimated
			Yes	62.673	39	.009	.999	.010	.012				
Negative	Warmth and	CLPM	No	2,368.547	27	<.001	.862	.139	.089	45.365	18	<.001	Freely

contact	anger		Yes	2,429.394	45	<.001	.861	.108	.089				estimated
		RI-CLPM	No	45.230	21	.002	.999	.015	.010	50.377	18	<.001	Freely estimated
			Yes	95.937	39	<.001	.997	.017	.016				
Positive and negative contact	Warmth and anger	CLPM	No	3,048.269	48	<.001	.882	.116	.076	56.228	32	.005	Freely estimated
			Yes	3,106.609	80	<.001	.882	.090	.077				
		RI-CLPM	No	62.080	38	.008	.999	.011	.009	62.524	32	<.001	Freely estimated
			Yes	124.874	70	<.001	.998	.013	.015				
Barlow et al. (2019) – Non-Pacific Islander sample													
Positive contact	Warmth	CLPM	No	1,987.023	12	<.001	.850	.187	.097	15.223	8	.055	Stationarity
			Yes	2,068.532	20	<.001	.850	.145	.097				
		RI-CLPM	No	28.492	9	.001	.999	.020	.011	13.156	8	.107	Stationarity
			Yes	41.488	17	.001	.998	.017	.016				
Negative contact	Warmth	CLPM	No	1,959.524	12	<.001	.795	.185	.096	10.399	8	.238	Stationarity
			Yes	1,997.433	20	<.001	.796	.143	.097				
		RI-CLPM	No	33.029	9	<.001	.998	.023	.011	9.447	8	.306	Stationarity
			Yes	41.780	17	.001	.998	.017	.013				
Positive and negative contact	Warmth	CLPM	No	2,748.728	27	<.001	.846	.143	.081	28.986	18	.049	Freely estimated
			Yes	2,813.362	45	<.001	.845	.111	.082				
		RI-CLPM	No	64.560	21	<.001	.998	.019	.012	23.827	18	.161	Stationarity
			Yes	87.483	39	<.001	.997	.015	.015				
Positive contact	Anger	CLPM	No	1,808.086	12	<.001	.806	.171	.090	21.033	8	.007	Freely estimated
			Yes	1,881.229	20	<.001	.805	.133	.090				
		RI-CLPM	No	23.055	9	.006	.999	.015	.010	16.092	8	.041	Freely

			Yes	39.091	17	.002	.998	.015	.015				estimated
Negative contact	Anger	CLPM	No	1,414.508	12	<.001	.803	.154	.095	18.400	8	.018	Freely estimated
			Yes	1,451.633	20	<.001	.802	.119	.096				
		RI-CLPM	No	20.294	9	.016	.999	.015	.008	25.890	8	.001	Freely estimated
			Yes	46.365	17	<.001	.996	.018	.012				
Positive and negative contact	Anger	CLPM	No	2,509.864	27	<.001	.820	.133	.080	41.582	18	.001	Freely estimated
			Yes	2,582.791	45	<.001	.819	.104	.081				
		RI-CLPM	No	51.883	21	<.001	.998	.015	.010	45.979	18	<.001	Freely estimated
			Yes	97.894	39	<.001	.996	.017	.015				
Positive contact	Warmth and anger	CLPM	No	2,628.219	27	<.001	.866	.139	.084	29.730	18	.040	Freely estimated
			Yes	2,706.667	45	<.001	.865	.108	.084				
		RI-CLPM	No	44.204	21	.002	.999	.014	.009	23.719	18	.164	Stationarity
			Yes	67.788	39	.003	.999	.012	.013				
Negative contact	Warmth and anger	CLPM	No	2,484.884	27	<.001	.846	.135	.087	30.718	18	.031	Freely estimated
			Yes	2,531.843	45	<.001	.846	.105	.087				
		RI-CLPM	No	51.779	21	<.001	.998	.017	.012	35.184	18	.009	Freely estimated
			Yes	86.734	39	<.001	.997	.015	.013				
Positive and negative contact	Warmth and anger	CLPM	No	3,279.771	48	<.001	.868	.115	.075	57.378	32	.004	Freely estimated
			Yes	3,365.428	80	<.001	.867	.089	.076				
		RI-CLPM	No	84.769	38	<.001	.998	.015	.011	54.081	32	.009	Freely estimated
			Yes	138.523	70	<.001	.997	.014	.014				
Bohman & Miklikowska (2021)													
Intergroup	Attitudes	CLPM	No	153.116	24	<.001	.950	.090	.062	138.160	12	<.001	Freely

Friendships			Yes	291.277	36	<.001	.901	.104	.103				estimated
		RI-CLPM	No	70.600	21	<.001	.981	.060	.032	146.080	12	<.001	Freely estimated
			Yes	216.684	33	<.001	.929	.092	.105				
Bohrer et al. (2019)													
Positive Contact	Attitudes	CLPM	No	270.721	12	<.001	.860	.226	.088	12.563	8	.128	Stationarity
			Yes	308.087	20	<.001	.859	.175	.092				
		RI-CLPM	No	27.682	9	.001	.992	.061	.033	9.179	8	.327	Stationarity
			Yes	35.709	17	.005	.992	.046	.037				
Bracegirdle et al. (2022) – Asian sample													
Intergroup Friendships	Attitudes	CLPM	No	304.136	24	<.001	.873	.170	.091	26.056	12	.011	Freely estimated
			Yes	320.830	36	<.001	.867	.142	.099				
		RI-CLPM	No	47.049	21	.001	.991	.050	.039	11.444	12	.491	Stationarity
			Yes	56.143	33	.007	.991	.039	.050				
Bracegirdle et al. (2022) – White British sample													
Intergroup Friendships	Attitudes	CLPM	No	118.135	24	<.001	.897	.154	.083	17.293	12	.139	Stationarity
			Yes	136.105	36	<.001	.895	.127	.097				
		RI-CLPM	No	45.045	21	.002	.979	.074	.058	7.426	12	.828	Stationarity
			Yes	50.222	33	.028	.987	.046	.063				
Cernat (2017) – Parent sample													
Positive contact	Emotions	CLPM	No	50.036	12	<.001	.917	.120	.065	42.618	8	<.001	Freely estimated
			Yes	92.194	20	<.001	.849	.126	.084				
		RI-CLPM	No	15.925	9	.068	.988	.052	.031	52.791	8	<.001	Freely estimated
			Yes	70.570	17	<.001	.896	.113	.077				

Negative contact	Emotions	CLPM	No	35.396	12	<.001	.944	.091	.053	39.679	8	<.001	Freely estimated
			Yes	75.970	20	<.001	.860	.111	.078				
		RI-CLPM	No	15.470	9	.079	.987	.051	.032	52.885	8	<.001	Freely estimated
			Yes	68.145	17	<.001	.878	.112	.070				
Positive and negative contact	Emotions	CLPM	No	76.394	27	<.001	.934	.088	.052	69.847	18	<.001	Freely estimated
			Yes	146.307	45	<.001	.864	.099	.075				
		RI-CLPM	No	37.990	21	.013	.982	.052	.039	76.351	18	<.001	Freely estimated
			Yes	118.411	39	<.001	.899	.091	.073				
Positive contact	Stereotypes	CLPM	No	33.349	12	.001	.944	.088	.052	30.824	8	<.001	Freely estimated
			Yes	64.538	20	<.001	.881	.100	.077				
		RI-CLPM	No	12.392	9	.192	.993	.037	.027	32.279	8	<.001	Freely estimated
			Yes	46.474	17	<.001	.924	.087	.074				
Negative contact	Stereotypes	CLPM	No	16.124	12	.186	.987	.039	.034	14.916	8	.061	Stationarity
			Yes	31.651	20	.047	.959	.052	.056				
		RI-CLPM	No	14.748	9	.098	.983	.051	.029	14.821	8	.063	Stationarity
			Yes	29.786	17	.028	.956	.059	.053				
Positive and negative contact	Stereotypes	CLPM	No	56.959	27	.001	.954	.068	.043	45.847	18	<.001	Freely estimated
			Yes	103.203	45	<.001	.907	.075	.067				
		RI-CLPM	No	31.563	21	.065	.986	.042	.035	46.509	18	<.001	Freely estimated
			Yes	79.857	39	<.001	.937	.067	.067				
Positive contact	Emotions and stereotypes	CLPM	No	63.032	27	<.001	.943	.077	.051	76.532	18	<.001	Freely estimated
			Yes	139.558	45	<.001	.850	.096	.079				
		RI-CLPM	No	24.889	21	.252	.996	.023	.029	88.924	18	<.001	Freely

			Yes	115.624	39	<.001	.885	.090	.078				estimated
Negative contact	Emotions and stereotypes	CLPM	No	51.730	27	.003	.961	.062	.046	61.794	18	<.001	Freely estimated
			Yes	115.220	45	<.001	.882	.083	.071				
		RI-CLPM	No	25.056	21	.245	.995	.025	.030	77.732	18	<.001	Freely estimated
			Yes	105.535	39	<.001	.892	.085	.067				
Positive and negative contact	Emotions and stereotypes	CLPM	No	100.357	48	<.001	.947	.067	.045	108.180	32	<.001	Freely estimated
			Yes	209.437	80	<.001	.865	.083	.071				
		RI-CLPM	No	53.047	38	.053	.988	.036	.036	120.250	32	<.001	Freely estimated
			Yes	177.826	70	<.001	.892	.080	.071				
Cernat (2017) – Teenager sample													
Positive contact	Emotions	CLPM	No	56.785	12	<.001	.893	.132	.067	10.668	8	.221	Stationarit y
			Yes	69.084	20	<.001	.888	.105	.073				
		RI-CLPM	No	25.264	9	.003	.970	.080	.054	9.545	8	.298	Stationarit y
			Yes	33.623	17	.009	.966	.062	.058				
Negative contact	Emotions	CLPM	No	20.122	12	.065	.973	.055	.039	10.661	8	.222	Stationarit y
			Yes	30.823	20	.058	.965	.048	.052				
		RI-CLPM	No	10.547	9	.308	.997	.023	.027	12.176	8	.144	Stationarit y
			Yes	22.717	17	.159	.983	.036	.048				
Positive and negative contact	Emotions	CLPM	No	81.343	27	<.001	.913	.095	.051	24.820	18	.130	Stationarit y
			Yes	106.783	45	<.001	.903	.078	.060				
		RI-CLPM	No	42.645	21	.003	.973	.060	.047	25.338	18	.116	Stationarit y
			Yes	67.116	39	.003	.960	.053	.054				
Positive	Stereotypes	CLPM	No	62.621	12	<.001	.854	.147	.073	8.703	8	.368	Stationarit

contact			Yes	73.203	20	<.001	.853	.114	.081				y
		RI-CLPM	No	25.864	9	.002	.964	.084	.055	7.652	8	.468	Stationarity
			Yes	31.093	17	.019	.964	.062	.057				
Negative contact	Stereotypes	CLPM	No	22.248	12	.035	.954	.067	.049	13.895	8	.085	Stationarity
			Yes	36.131	20	.015	.929	.064	.064				
		RI-CLPM	No	4.705	9	.859	1.000	.000	.025	13.883	8	.085	Stationarity
			Yes	18.546	17	.355	.993	.021	.050				
Positive and negative contact	Stereotypes	CLPM	No	81.085	27	<.001	.903	.097	.055	29.013	18	.048	Freely estimated
			Yes	109.921	45	<.001	.884	.082	.067				
		RI-CLPM	No	37.183	21	.016	.978	.052	.046	26.287	18	.093	Stationarity
			Yes	62.773	39	.009	.961	.051	.056				
Positive contact	Emotions and stereotypes	CLPM	No	90.059	27	<.001	.886	.105	.061	21.840	18	.239	Stationarity
			Yes	113.251	45	<.001	.881	.083	.069				
		RI-CLPM	No	40.344	21	.007	.974	.057	.050	22.195	18	.223	Stationarity
			Yes	61.287	39	.013	.966	.048	.057				
Negative contact	Emotions and stereotypes	CLPM	No	46.567	27	.011	.955	.059	.043	23.948	18	.157	Stationarity
			Yes	70.717	45	.009	.945	.051	.056				
		RI-CLPM	No	17.374	21	.688	1.000	.000	.027	25.153	18	.121	Stationarity
			Yes	42.427	39	.326	.995	.016	.047				
Positive and negative contact	Emotions and stereotypes	CLPM	No	114.904	48	<.001	.915	.080	.049	45.306	32	.060	Stationarity
			Yes	160.836	80	<.001	.901	.067	.061				
		RI-CLPM	No	58.552	38	.018	.981	.043	.044	44.909	32	.065	Stationarity
			Yes	103.140	70	.006	.964	.043	.055				

Hässler et al. (2019)													
Cross-group friendships	Outgroup trust	CLPM	No	46.718	4	<.001	.913	.197	.049	9.568	4	.048	Freely estimated
			Yes	58.607	8	<.001	.903	.147	.058				
		RI-CLPM	No	0.899	1	.343	1.000	.000	.008	10.238	4	.037	Freely estimated
			Yes	11.167	5	.048	.992	.054	.032				
Jugert et al. (2011)													
Cross-group friendships	Affective attitudes	CLPM	No	19.784	4	.001	.905	.233	.053	12.994	4	.011	Freely estimated
			Yes	32.379	8	<.001	.852	.206	.089				
		RI-CLPM	No	0.002	1	.966	1.000	.000	.001	14.230	4	.007	Freely estimated
			Yes	15.025	5	.010	.967	.123	.075				
Cross-group friendships	Outgroup orientations	CLPM	No	26.196	4	<.001	.873	.286	.061	1.179	4	.882	Stationarity
			Yes	28.527	8	<.001	.887	.190	.065				
		RI-CLPM	No	0.317	1	.574	1.000	.000	.009	3.355	4	.500	Stationarity
			Yes	3.753	5	.585	1.000	.000	.035				
Cross-group friendships	Affective attitudes and outgroup orientations	CLPM	No	32.593	9	<.001	.919	.186	.053	19.423	9	.022	Freely estimated
			Yes	51.697	18	<.001	.884	.157	.080				
		RI-CLPM	No	2.018	3	.569	1.000	.000	.018	15.536	9	.077	Stationarity
			Yes	17.787	12	.122	.993	.047	.052				
Kotzur & Wagner (2021)													
Positive contact	Prejudice	CLPM	No	86.853	4	<.001	.949	.207	.036	14.870	4	.005	Freely estimated
			Yes	105.513	8	<.001	.944	.153	.041				
		RI-CLPM	No	0.100	1	.752	1.000	.000	.002	30.979	4	<.001	Freely estimated
			Yes	28.546	5	<.001	.992	.073	.031				

Negative contact	Prejudice	CLPM	No	67.799	4	<.001	.950	.201	.038	3.261	4	.515	Stationarity
			Yes	68.183	8	<.001	.951	.141	.040				
		RI-CLPM	No	0.234	1	.628	1.000	.000	.005	3.159	4	.532	Stationarity
			Yes	3.424	5	.635	1.000	.000	.017				
Positive and negative contact	Prejudice	CLPM	No	102.811	9	<.001	.960	.148	.035	16.078	9	.065	Stationarity
			Yes	116.596	18	<.001	.958	.108	.039				
		RI-CLPM	No	0.908	3	.823	1.000	.000	.004	25.169	9	.003	Freely estimated
			Yes	26.429	12	.009	.995	.044	.025				
Levin et al. (2003)													
Outgroup friendships	Intergroup anxiety	CLPM	No	366.927	12	<.001	.848	.181	.085	12.575	8	.127	Stationarity
			Yes	371.514	20	<.001	.848	.140	.089				
		RI-CLPM	No	10.084	9	.344	1.000	.011	.013	6.984	8	.538	Stationarity
			Yes	16.983	17	.456	1.000	.000	.020				
Outgroup friendships	Ingroup bias	CLPM	No	274.249	12	<.001	.869	.175	.079	12.643	8	.125	Stationarity
			Yes	283.906	20	<.001	.869	.135	.083				
		RI-CLPM	No	9.992	9	.351	1.000	.005	.016	5.799	8	.670	Stationarity
			Yes	15.403	17	.567	1.000	.000	.022				
Outgroup friendships	Intergroup anxiety and ingroup bias	CLPM	No	437.928	27	<.001	.864	.138	.070	24.245	18	.147	Stationarity
			Yes	453.826	45	<.001	.865	.107	.074				
		RI-CLPM	No	21.441	21	.432	1.000	.000	.016	10.636	18	.909	Stationarity
			Yes	31.468	39	.799	1.000	.000	.020				
Levin et al. (2007)													
Outgroup	Intergroup	CLPM	No	321.723	12	<.001	.775	.181	.094	77.989	8	<.001	Freely

dating	anxiety		Yes	402.187	20	<.001	.730	.153	.105				estimated
		RI-CLPM	No	15.659	9	.074	.995	.030	.020	154.410	8	<.001	Freely estimated
			Yes	172.771	17	<.001	.904	.099	.072				
Outgroup dating	Ingroup bias	CLPM	No	250.346	12	<.001	.814	.174	.089	59.211	8	<.001	Freely estimated
			Yes	306.064	20	<.001	.774	.148	.101				
		RI-CLPM	No	14.819	9	.096	.996	.030	.022	113.690	8	<.001	Freely estimated
			Yes	138.583	17	<.001	.914	.099	.073				
Outgroup dating	Intergroup anxiety and ingroup bias	CLPM	No	422.182	27	<.001	.823	.139	.075	84.121	18	<.001	Freely estimated
			Yes	502.939	45	<.001	.797	.115	.084				
		RI-CLPM	No	28.416	21	.129	.997	.019	.020	141.000	18	<.001	Freely estimated
			Yes	178.400	39	<.001	.946	.064	.054				
Northcutt, Bohmert & DeMaris (2015)													
Interracial friendships	Affirmative action	CLPM	No	58.492	12	<.001	.936	.122	.063	10.008	8	.265	Stationarity
			Yes	68.186	20	<.001	.934	.096	.071				
		RI-CLPM	No	14.532	9	.105	.994	.043	.031	15.932	8	.043	Freely estimated
			Yes	30.541	17	.023	.985	.050	.043				
Interracial friendships	Feeling of commonality	CLPM	No	44.529	12	<.001	.907	.099	.060	16.002	8	.042	Freely estimated
			Yes	58.906	20	<.001	.881	.087	.072				
		RI-CLPM	No	7.032	9	.634	1.000	.000	.021	13.665	8	.091	Stationarity
			Yes	21.395	17	.209	.986	.032	.047				
Interracial friendships	Affirmative action and	CLPM	No	79.673	27	<.001	.944	.085	.051	27.721	18	.066	Stationarity
			Yes	106.345	45	<.001	.934	.072	.062				

	feeling of commonality	RI-CLPM	No	22.364	21	.379	1.000	.000	.027	30.181	18	.036	Freely estimated
			Yes	52.762	39	.070	.989	.032	.044				
Stark (2015)													
Cross-group friendships	Prejudice	CLPM	No	99.335	4	<.001	.863	.229	.073	14.095	4	.007	Freely estimated
			Yes	113.430	8	<.001	.848	.171	.079				
		RI-CLPM	No	31.959	1	<.001	.955	.261	.064	35.900	4	<.001	Freely estimated
			Yes	67.860	5	<.001	.909	.167	.070				
Swart et al. (2011)													
Cross-group friendships	Outgroup attitudes	CLPM	No	58.840	4	<.001	.846	.224	.067	13.166	4	.010	Freely estimated
			Yes	76.609	8	<.001	.832	.165	.078				
		RI-CLPM	No	2.108	1	.147	.998	.050	.014	41.224	4	<.001	Freely estimated
			Yes	40.064	5	<.001	.961	.100	.048				
Cross-group friendships	Outgroup variability	CLPM	No	42.924	4	<.001	.890	.185	.051	1.094	4	.895	Stationarit y
			Yes	46.385	8	<.001	.898	.127	.052				
		RI-CLPM	No	4.136	1	.042	.993	.091	.020	3.703	4	.448	Stationarit y
			Yes	7.597	5	.180	.995	.035	.030				
Cross-group friendships	Negative action tendencies	CLPM	No	56.508	4	<.001	.868	.227	.061	1.334	4	.856	Stationarit y
			Yes	60.089	8	<.001	.874	.156	.063				
		RI-CLPM	No	2.233	1	.135	.998	.058	.014	1.657	4	.799	Stationarit y
			Yes	3.615	5	.606	1.000	.000	.020				
Cross-group friendships	Outgroup attitudes,	CLPM	No	104.464	16	<.001	.875	.139	.057	16.436	16	.423	Stationarit y
			Yes	120.264	32	<.001	.878	.097	.063				

	outgroup variability and negative action tendencies	RI-CLPM	No	9.810	6	.133	.996	.041	.018	25.762	16	.057	Stationarity
			Yes	35.567	22	.034	.988	.036	.036				
Van Laar et al. (2005) – Non-Asian sample													
Intergroup roommates	Outgroup attitudes	CLPM	No	126.823	12	<.001	.886	.128	.051	21.316	8	.006	Freely estimated
			Yes	131.376	20	<.001	.876	.104	.060				
		RI-CLPM	No	37.649	9	<.001	.970	.076	.028	11.801	8	.160	Stationarity
			Yes	43.535	17	<.001	.968	.057	.039				
Intergroup roommates	Intergroup unease	CLPM	No	77.564	12	<.001	.803	.101	.055	33.034	8	<.001	Freely estimated
			Yes	106.957	20	<.001	.734	.091	.070				
		RI-CLPM	No	13.958	9	.124	.995	.019	.018	15.640	8	.048	Freely estimated
			Yes	30.222	17	.025	.969	.034	.035				
Intergroup roommates	Multicultural competence	CLPM	No	94.021	12	<.001	.789	.111	.062	17.695	8	.024	Freely estimated
			Yes	101.304	20	<.001	.776	.089	.069				
		RI-CLPM	No	15.436	9	.080	.993	.023	.021	9.222	8	.324	Stationarity
			Yes	23.747	17	.126	.994	.015	.029				
Intergroup roommates	Outgroup attitudes, intergroup unease and multicultural competence	CLPM	No	256.740	48	<.001	.863	.092	.055	65.302	32	<.001	Freely estimated
			Yes	316.326	80	<.001	.847	.075	.063				
		RI-CLPM	No	67.967	38	.002	.983	.036	.026	47.831	32	.036	Freely estimated
			Yes	115.050	70	.001	.974	.033	.034				
Van Laar et al. (2005) – Non-Caucasian sample													
Intergroup	Outgroup	CLPM	No	141.751	12	<.001	.876	.129	.061	35.753	8	<.001	Freely

roommates	attitudes		Yes	172.419	20	<.001	.857	.107	.072				estimated
		RI-CLPM	No	12.399	9	.192	.997	.025	.017	31.234	8	<.001	Freely estimated
			Yes	46.106	17	<.001	.980	.044	.040				
Intergroup roommates	Intergroup unease	CLPM	No	130.127	12	<.001	.788	.119	.065	33.335	8	<.001	Freely estimated
			Yes	154.892	20	<.001	.743	.101	.077				
		RI-CLPM	No	14.191	9	.116	.993	.024	.021	26.967	8	<.001	Freely estimated
			Yes	43.868	17	<.001	.958	.044	.041				
Intergroup roommates	Multicultural competence	CLPM	No	122.779	12	<.001	.804	.120	.068	26.931	8	<.001	Freely estimated
			Yes	142.972	20	<.001	.779	.099	.076				
		RI-CLPM	No	5.186	9	.818	1.000	.000	.011	21.676	8	.006	Freely estimated
			Yes	29.951	17	.029	.989	.024	.032				
Intergroup roommates	Outgroup attitudes, intergroup unease and multicultural competence	CLPM	No	304.036	48	<.001	.885	.088	.057	75.433	32	<.001	Freely estimated
			Yes	373.206	80	<.001	.872	.071	.063				
		RI-CLPM	No	39.789	38	.390	1.000	.000	.017	66.493	32	<.001	Freely estimated
			Yes	108.947	70	.002	.989	.022	.030				
Van Laar et al. (2005) – Non-Latino sample													
Intergroup roommates	Outgroup attitudes	CLPM	No	128.875	12	<.001	.882	.126	.055	64.483	8	<.001	Freely estimated
			Yes	188.182	20	<.001	.848	.111	.073				
		RI-CLPM	No	27.517	9	.001	.992	.038	.021	33.396	8	<.001	Freely estimated
			Yes	64.101	17	<.001	.974	.049	.042				

Intergroup roommates	Intergroup unease	CLPM	No	71.525	12	<.001	.863	.087	.046	65.948	8	<.001	Freely estimated
			Yes	143.320	20	<.001	.754	.090	.072				
		RI-CLPM	No	20.040	9	.018	.991	.026	.018	35.227	8	<.001	Freely estimated
			Yes	61.746	17	<.001	.937	.050	.043				
Intergroup roommates	Multicultural competence	CLPM	No	120.474	12	<.001	.787	.127	.065	60.080	8	<.001	Freely estimated
			Yes	175.479	20	<.001	.725	.111	.082				
		RI-CLPM	No	16.198	9	.063	.996	.020	.017	32.383	8	<.001	Freely estimated
			Yes	54.140	17	<.001	.966	.043	.041				
Intergroup roommates	Outgroup attitudes, intergroup unease and multicultural competence	CLPM	No	281.700	48	<.001	.894	.087	.051	122.330	32	<.001	Freely estimated
			Yes	399.377	80	<.001	.875	.073	.060				
		RI-CLPM	No	54.496	38	.040	.997	.017	.018	78.951	32	<.001	Freely estimated
			Yes	137.471	70	<.001	.985	.027	.029				
Van Zalk et al. (2013)													
Outgroup friendships	Tolerance	CLPM	No	41.406	4	<.001	.943	.157	.042	21.106	4	<.001	Freely estimated
			Yes	65.470	8	<.001	.930	.123	.051				
		RI-CLPM	No	7.760	1	.005	.989	.136	.019	30.346	4	<.001	Freely estimated
			Yes	38.104	5	<.001	.967	.107	.046				

Outgroup friendships	Xenophobia	CLPM	No	50.517	4	<.001	.922	.172	.049	17.721	4	.001	Freely estimated
			Yes	72.297	8	<.001	.912	.130	.053				
		RI-CLPM	No	5.478	1	.019	.992	.109	.016	28.778	4	<.001	Freely estimated
			Yes	34.195	5	<.001	.971	.094	.040				
Outgroup friendships	Tolerance and xenophobia	CLPM	No	53.386	9	<.001	.966	.110	.039	25.019	9	.003	Freely estimated
			Yes	79.447	18	<.001	.958	.086	.044				
		RI-CLPM	No	11.736	3	.008	.993	.086	.021	37.232	9	<.001	Freely estimated
			Yes	48.986	12	<.001	.980	.072	.037				
Vezzali et al. (2022) – Immigrant sample													
Contact quantity	Positive outgroup attitudes	CLPM	No	63.250	12	<.001	.884	.162	.073	11.121	8	.195	Stationarity
			Yes	75.369	20	<.001	.880	.128	.081				
		RI-CLPM	No	28.118	9	.001	.969	.097	.058	30.057	8	<.001	Freely estimated
			Yes	59.092	17	<.001	.914	.117	.074				
Contact quality	Positive outgroup attitudes	CLPM	No	86.092	12	<.001	.750	.201	.104	13.473	8	.097	Stationarity
			Yes	100.430	20	<.001	.734	.161	.112				
		RI-CLPM	No	16.750	9	.053	.978	.069	.050	34.105	8	<.001	Freely estimated
			Yes	52.523	17	<.001	.889	.113	.075				

Contact quantity and contact quality	Positive outgroup attitudes	CLPM	No	137.994	27	<.001	.845	.160	.082	40.063	18	.002	Freely estimated
			Yes	179.615	45	<.001	.818	.134	.093				
		RI-CLPM	No	49.145	21	<.001	.970	.080	.054	76.428	18	<.001	Freely estimated
			Yes	129.215	39	<.001	.885	.115	.075				
Contact quantity	Negative outgroup attitudes	CLPM	No	48.879	12	<.001	.892	.143	.073	17.049	8	.030	Freely estimated
			Yes	65.694	20	<.001	.870	.121	.088				
		RI-CLPM	No	16.042	9	.066	.982	.067	.038	17.781	8	.023	Freely estimated
			Yes	34.027	17	.008	.951	.081	.073				
Contact quality	Negative outgroup attitudes	CLPM	No	84.647	12	<.001	.719	.192	.108	16.520	8	.036	Freely estimated
			Yes	103.314	20	<.001	.691	.156	.115				
		RI-CLPM	No	13.620	9	.137	.985	.051	.047	31.693	8	<.001	Freely estimated
			Yes	45.765	17	<.001	.899	.097	.071				
Contact quantity and contact quality	Negative outgroup attitudes	CLPM	No	118.904	27	<.001	.845	.149	.083	48.652	18	<.001	Freely estimated
			Yes	170.303	45	<.001	.802	.130	.096				
		RI-CLPM	No	35.718	21	.024	.981	.059	.044	66.138	18	<.001	Freely estimated
			Yes	104.560	39	<.001	.898	.100	.072				
Contact	Positive and	CLPM	No	107.208	27	<.001	.862	.133	.066	39.506	18	.002	Freely

quantity	negative outgroup attitudes		Yes	147.870	45	<.001	.830	.115	.084				estimated
		RI-CLPM	No	52.055	21	<.001	.959	.083	.048	50.179	18	<.001	Freely estimated
			Yes	102.939	39	<.001	.899	.095	.070				
Contact quality	Positive and negative outgroup attitudes	CLPM	No	126.260	27	<.001	.773	.150	.086	39.105	18	.003	Freely estimated
			Yes	166.074	45	<.001	.729	.127	.100				
		RI-CLPM	No	34.321	21	.033	.976	.056	.046	58.551	18	<.001	Freely estimated
			Yes	94.823	39	<.001	.884	.090	.074				
Contact quantity and contact quality	Positive and negative outgroup attitudes	CLPM	No	188.778	48	<.001	.840	.133	.073	76.934	32	<.001	Freely estimated
			Yes	267.071	80	<.001	.794	.117	.090				
		RI-CLPM	No	80.469	38	<.001	.962	.072	.050	105.670	32	<.001	Freely estimated
			Yes	189.693	70	<.001	.876	.097	.073				
Vezzali et al. (2022) – Italian sample													
Contact quantity	Positive outgroup attitudes	CLPM	No	110.201	12	<.001	.893	.158	.079	16.781	8	.032	Freely estimated
			Yes	127.525	20	<.001	.885	.127	.083				
		RI-CLPM	No	22.487	9	.007	.987	.064	.041	27.825	8	<.001	Freely estimated
			Yes	50.554	17	<.001	.967	.074	.049				
Contact	Positive	CLPM	No	138.802	12	<.001	.869	.184	.099	24.976	8	.002	Freely

quality	outgroup attitudes		Yes	165.010	20	<.001	.852	.151	.099				estimated
		RI-CLPM	No	20.342	9	.016	.990	.059	.026	32.212	8	<.001	Freely estimated
			Yes	53.753	17	<.001	.965	.080	.040				
Contact quantity and contact quality	Positive outgroup attitudes	CLPM	No	231.722	27	<.001	.883	.150	.080	33.407	18	.015	Freely estimated
			Yes	263.564	45	<.001	.875	.121	.083				
		RI-CLPM	No	58.020	21	<.001	.982	.067	.037	63.592	18	<.001	Freely estimated
			Yes	122.928	39	<.001	.955	.078	.047				
Contact quantity	Negative outgroup attitudes	CLPM	No	87.790	12	<.001	.888	.136	.065	18.446	8	.018	Freely estimated
			Yes	103.120	20	<.001	.872	.112	.074				
		RI-CLPM	No	13.615	9	.137	.994	.035	.026	14.503	8	.070	Stationarity
			Yes	28.392	17	.041	.984	.043	.041				
Contact quality	Negative outgroup attitudes	CLPM	No	97.369	12	<.001	.885	.149	.082	16.177	8	.040	Freely estimated
			Yes	109.941	20	<.001	.873	.121	.086				
		RI-CLPM	No	10.885	9	.284	.998	.022	.024	9.662	8	.290	Stationarity
			Yes	20.545	17	.247	.995	.025	.036				
Contact quantity and contact	Negative outgroup attitudes	CLPM	No	183.858	27	<.001	.892	.131	.070	31.694	18	.024	Freely estimated
			Yes	211.664	45	<.001	.884	.105	.075				

quality		RI-CLPM	No	32.703	21	.050	.994	.036	.029	42.827	18	<.001	Freely estimated
			Yes	77.744	39	<.001	.974	.053	.043				
Contact quantity	Positive and negative outgroup attitudes	CLPM	No	164.210	27	<.001	.894	.122	.065	35.685	18	.008	Freely estimated
			Yes	197.351	45	<.001	.881	.101	.072				
		RI-CLPM	No	38.338	21	.012	.988	.047	.037	52.054	18	<.001	Freely estimated
			Yes	91.386	39	<.001	.962	.061	.051				
Contact quality	Positive and negative outgroup attitudes	CLPM	No	199.746	27	<.001	.884	.137	.078	44.717	18	<.001	Freely estimated
			Yes	239.941	45	<.001	.864	.114	.081				
		RI-CLPM	No	40.663	21	.006	.988	.050	.026	53.267	18	<.001	Freely estimated
			Yes	95.587	39	<.001	.963	.064	.043				
Contact quantity and contact quality	Positive and negative outgroup attitudes	CLPM	No	301.972	48	<.001	.889	.123	.069	57.12	32	.004	Freely estimated
			Yes	354.073	80	<.001	.878	.100	.074				
		RI-CLPM	No	80.830	38	<.001	.983	.054	.037	100.050	32	<.001	Freely estimated
			Yes	183.421	70	<.001	.952	.067	.048				
Wagner et al. (2008)													
Positive contact	Prejudice	CLPM	No	140.333	4	<.001	.947	.231	.035	3.106	4	.540	Stationarit y
			Yes	157.425	8	<.001	.947	.163	.036				

		RI-CLPM	No	1.254	1	.263	1.000	.015	.006	1.183	4	.881	Stationarit y
			Yes	2.418	5	.789	1.000	.000	.009				
Wölfer & Hewstone (2018)													
Outgroup friends	Attitudes	CLPM	No	159.710	4	<.001	.946	.199	.039	10.802	4	.029	Freely estimated
			Yes	166.510	8	<.001	.943	.143	.041				
		RI-CLPM	No	7.512	1	.006	.999	.063	.012	31.465	4	<.001	Freely estimated
			Yes	39.079	5	<.001	.991	.073	.035				
Wölfer et al. (2016) – Cohort 1													
Cross-group friendships	Tolerant attitudes	CLPM	No	1,780.323	12	<.001	.682	.349	.120	17.832	8	.023	Freely estimated
			Yes	1,798.155	20	<.001	.680	.271	.122				
		RI-CLPM	No	372.804	9	<.001	.935	.183	.218	876.000	8	<.001	Freely estimated
			Yes	1,248.801	17	<.001	.778	.245	.260				
Wölfer et al. (2016) – Cohort 2													
Cross-group friendships	Tolerant attitudes	CLPM	No	2,240.998	12	<.001	.583	.392	.143	137.970	8	<.001	Freely estimated
			Yes	2,378.964	20	<.001	.559	.313	.163				
		RI-CLPM	No	492.717	9	<.001	.910	.211	.319	1,134.200	8	<.001	Freely estimated
			Yes	1,626.886	17	<.001	.699	.280	.143				

Wölfer et al. (2019)												
Direct Contact	Intergroup anxiety	CLPM	No	186.320	4	<.001	.885	.249	.068	4.603	4	.331
			Yes	190.922	8	<.001	.885	.176	.070			
		RI-CLPM	No	0.645	1	.422	1.000	.000	.006	7.740	4	.102
			Yes	8.385	5	.136	.998	.030	.020			

Notes. Df = degrees of freedom, p = p-value, CFI = Comparative Fit Index, RMSEA = Root Mean Square Error of Approximation, SRMR = Standardized Root Mean Square Residual. For the stationarity model, we implemented equality constraints for identical stability (i.e, contactT1 → contactT2, attitudesT1 → attitudesT2, etc.) and cross-lagged (i.e., contactT1 → attitudesT2, attitudesT1 → contactT1, etc.) coefficients across measurement intervals. We did not report the model fit of the FF-CLPM, the the model is just-identified by nature (i.e., df = 0), resulting in perfect model fit.

Supplementary Table 4: Power Analysis

Table 4.1

Power analysis of the Cross-Lagged Panel Model (CLPM)

N	Number of waves	Size of longitudinal contact-attitudes effect (beta)	Power (1 – β)
100	3	.03	.08
		.04	.08
		.05	.11
		.06	.12
	4	.03	.06
		.04	.08
		.05	.09
		.06	.11
	5	.03	.08
		.04	.09
		.05	.10
		.06	.12
200	3	.03	.09
		.04	.11
		.05	.14
		.06	.18
	4	.03	.08
		.04	.10
		.05	.12
		.06	.16
	5	.03	.09
		.04	.11
		.05	.13
		.06	.17
300	3	.03	.10
		.04	.13

		.05	.18
		.06	.24
	4	.03	.10
		.04	.13
		.05	.16
		.06	.22
	5	.03	.09
		.04	.13
		.05	.18
		.06	.24
500	3	.03	.12
		.04	.18
		.05	.25
		.06	.35
	4	.03	.11
		.04	.18
		.05	.24
		.06	.32
	5	.03	.12
		.04	.18
		.05	.26
		.06	.34
800	3	.03	.17
		.04	.28
		.05	.38
		.06	.50
	4	.03	.16
		.04	.25
		.05	.38
		.06	.51
	5	.03	.17
		.04	.27

		.05	.39
		.06	.52
1,000	3	.03	.20
		.04	.32
		.05	.45
		.06	.59
	4	.03	.21
		.04	.31
		.05	.43
		.06	.57
	5	.03	.20
		.04	.32
		.05	.45
		.06	.60
2,000	3	.03	.34
		.04	.53
		.05	.73
		.06	.87
	4	.03	.35
		.04	.52
		.05	.72
		.06	.87
	5	.03	.34
		.04	.55
		.05	.74
		.06	.88

Note. This a posteriori power analysis was conducted with the R-based pwrsem-tool[1]. We ran a bivariate (i.e., simulating only one contact and one attitude construct) (FF-)CLPM with stationarity assumptions (as the powRICLPM-tool, which we used to calculate the within-person longitudinal contact-attitude effects in the RI-CLPM, does not permit more than two variables). The power was estimated using the pwrSEM shiny app with 1,000 simulations, alpha level = .05. The power was averaged across longitudinal contact-attitude effects in the different measurement waves. As further parameters, we chose: the contact stability effect: beta = .45, the attitudes stability effect: beta = .47, the longitudinal attitudes-contact effect: beta = .05, the (residual) correlation between contact and attitude at the same measurement wave: r = .08, contact (residual) variance t1: s^2 = 1.00, t2: s^2 = .68,

t3: s^2 = .63, t4: s^2 = .71, t5: s^2 = .50, attitude (residual) variance t1: s^2 = 1.00, t2: s^2 = .70, t3: s^2 = .69, t4: s^2 = .66, t5: s^2 = .55. These parameters were chosen based on an average of a random selection of effects found in this re-analysis (one bivariate analysis per publication that was analysed) using non-stationary models and the smallest observed parameters. Thus, the parameters represent conservative, but plausible values.

Table 4.2

Power analysis of the Full-Forward Cross-Lagged Panel Model (FF-CLPM)

N	Number of waves	Size of longitudinal contact-attitudes effect (beta)	Power (1 – β)
100	3	.02	.05
		.03	.06
		.04	.07
		.05	.09
	4	.02	.07
		.03	.07
		.04	.08
		.05	.10
	5	.02	.07
		.03	.08
		.04	.09
		.05	.12
200	3	.02	.07
		.03	.09
		.04	.10
		.05	.13
	4	.02	.06
		.03	.06
		.04	.09
		.05	.11
	5	.02	.08
		.03	.10
		.04	.11
		.05	.13
300	3	.02	.07
		.03	.09
		.04	.12
		.05	.14

	4	.02	.09
		.03	.07
		.04	.09
		.05	.13
	5	.02	.08
		.03	.10
		.04	.14
		.05	.18
500	3	.02	.06
		.03	.08
		.04	.13
		.05	.19
	4	.02	.07
		.03	.11
		.04	.15
		.05	.21
	5	.02	.09
		.03	.13
		.04	.19
		.05	.26
800	3	.02	.10
		.03	.15
		.04	.21
		.05	.30
	4	.02	.10
		.03	.14
		.04	.21
		.05	.29
	5	.02	.11
		.03	.17
		.04	.26
		.05	.36

1,000	3	.02	.10
		.03	.15
		.04	.25
		.05	.36
	4	.02	.09
		.03	.16
		.04	.25
		.05	.35
	5	.02	.12
		.03	.18
		.04	.29
		.05	.42
2,000	3	.02	.12
		.03	.26
		.04	.44
		.05	.62
	4	.02	.12
		.03	.26
		.04	.41
		.05	.58
	5	.02	.20
		.03	.34
		.04	.54
		.05	.75

Note. This a posteriori power analysis was conducted with the R-based pwrsem-tool[1]. We ran a bivariate (i.e., simulating only one contact and one attitude construct) (FF-)CLPM with stationarity assumptions (as the powRICLPM-tool, which we used to calculate the within-person longitudinal contact-attitude effects in the RI-CLPM, does not permit more than two variables). The power was estimated using the pwrSEM shiny app with 1,000 simulations, alpha level = .05. The power estimation is based on the longitudinal contact-attitude effect in the last measurement interval. As further parameters, we chose: the lag-1 contact stability effect: beta = .31, the lag-1 attitudes stability effect: beta = .35, the longitudinal lag-1 attitudes-contact effect: beta = .05, the (residual) correlation between contact and attitudes at the same measurement wave: r = .09, contact (residual) variance t1: s^2 = 1.00, t2: s^2 = .69, t3: s^2 = .58, t4: s^2 = .65, t5: s^2 = .44, attitudes (residual) variance t1: s^2 = 1.00, t2: s^2 = .70, t3: s^2 = .65, t4: s^2 = .58, t5: s^2 = .50. The higher-lag effects were chosen as follows: lag-2 contact stability effect: beta = .20, lag-2 attitudes stability effect: beta = .21, longitudinal lag-2 contact-

attitudes effect: beta = .03, longitudinal lag-2 attitudes-contact effect: beta = .03; lag-3 contact stability effect: beta = .14, lag-3 attitudes stability effect: beta = .11, longitudinal lag-3 contact-attitudes effect: beta = .07, longitudinal lag-3 attitudes-contact effect: beta = .02; lag-4 contact stability effect: beta = .06, lag-4 attitudes stability effect: beta = .08, longitudinal lag-4 contact-attitudes effect: beta = .03, longitudinal lag-4 attitudes-contact effect: beta = .03. These parameters were chosen based on an average of a random selection of effects found in this re-analysis (one bivariate analysis per publication that was analysed) using non-stationary models and the smallest observed parameters. Thus, the parameters represent conservative, but plausible values.

Table 4.3

Power analysis of the Random-Intercept Cross-Lagged Panel Model (RI-CLPM)

N	Number of waves	Size of longitudinal within-person contact-attitudes effect (beta)	Power (1 – β)
100	3	.03	.05
		.04	.05
		.05	.07
		.06	.05
	4	.03	.07
		.04	.07
		.05	.07
		.06	.09
	5	.03	.07
		.04	.07
		.05	.06
		.06	.09
200	3	.03	.06
		.04	.07
		.05	.10
		.06	.08
	4	.03	.08
		.04	.09
		.05	.10
		.06	.11
	5	.03	.07
		.04	.08
		.05	.08
		.06	.10
300	3	.03	.08
		.04	.09
		.05	.09

		.06	.11
	4	.03	.08
		.04	.09
		.05	.11
		.06	.12
	5	.03	.09
		.04	.09
		.05	.11
		.06	.14
500	3	.03	.07
		.04	.08
		.05	.11
		.06	.14
	4	.03	.07
		.04	.10
		.05	.14
		.06	.17
	5	.03	.09
		.04	.11
		.05	.15
		.06	.20
800	3	.03	.09
		.04	.12
		.05	.17
		.06	.18
	4	.03	.11
		.04	.15
		.05	.21
		.06	.26
	5	.03	.09
		.04	.16
		.05	.22

		.06	.29
1,000	3	.03	.08
		.04	.12
		.05	.16
		.06	.21
	4	.03	.11
		.04	.15
		.05	.21
		.06	.31
	5	.03	.12
		.04	.19
		.05	.25
		.06	.33
2,000	3	.03	.16
		.04	.23
		.05	.29
		.06	.35
	4	.03	.16
		.04	.25
		.05	.39
		.06	.52
	5	.03	.20
		.04	.33
		.05	.44
		.06	.56

Note. This a posteriori power analysis was conducted with the R-package powRICLPM-tool[2]. We ran a bivariate (i.e., simulating only one contact and one attitudes construct) RI-CLPM (as the powRICLPM-tool does not permit more than two variables) with 1,000 simulations, alpha level = .05. As further parameters, we chose: the within-person contact stability effect: beta = .08, the within-person attitudes stability effect: beta = .12, the within-person longitudinal attitudes-contact effect: b = .05, the within-person (residual) correlation between contact and attitudes at the same measurement wave: r = .07, the between-person attitudes-contact correlation: r = .30, the share of between-person variance: 35%. These parameter choices are an average of a random selection of effects found in this re-analysis (one bivariate analysis per publication that was analysed) using non-stationary models and the smallest observed parameters. Thus, the parameters represent conservative, but plausible values.

References:

1. Wang, Y. A. & Rhemtulla, M. Power analysis for parameter estimation in structural equation modeling: A discussion and tutorial. Advances in Methods and Practices in Psychological Science **4**, 251524592091825 (2021).
2. Mulder, J. D. Power analysis for the random intercept cross-lagged panel model using the powRICLPM R-package. Structural Equation Modeling: A Multidisciplinary Journal **30**, 645–658 (2023).

Supplementary Table 5 – Results of the Internal Meta-Analysis without van Zalk et al. (2013) Data

Meta-Analytical Results of the Re-Analysis of Longitudinal Effect of Non-Valenced or Positive Intergroup Contact on Outgroup Attitudes

model	*k*	estimated average Fisher r-to-z transformed correlation coefficient	*p*	95% CI	test for heterogenity (*Q*)	*p* for heterogenity
CLPM freely estimated	161	0.0553 (0.0115)	<.001	[0.0328, 0.0778]	946.5304	<.001
FF-CLPM	57	0.0197 (0.0244)	.4181	[-0.0280, 0.0675]	562.2736	<.001
RI-CLPM freely estimated (between-person effect)	57	0.3243 (0.0528)	<.001	[0.2207, 0.4278]	9573.3920	<.001
RI-CLPM freely estimated (within-person effect)	161	0.0058 (0.0070)	.4061	[-0.0079, 0.0196]	534.6572	<.001

Notes. CLPM = Cross-Lagged Panel Model; FF-CLPM = Full-Forward Cross-Lagged Panel Model; RI-CLPM = Random-Intercept Cross-Lagged Panel Model. For all models, tests were nested within studies. For the free CLPM and free RI-CLPM within, timepoints were additionally nested in tests.

The data of van Zalk et al. (2013) contained only positive contact information; consequently, only the internal meta-analysis results for non-valenced or positive contact are reported. The difference in the results is negligible and the complete pattern of results including significance and valence of effects remains stable.
