# Supplementary material for: A comparative multi-method re-analysis of the longitudinal evidence for causal intergroup contact effects on attitudes
Source: Commun Psychol. 2026 Jul 15;4:107. doi: 10.1038/s44271-026-00495-8 (PMC13372666; doi:10.1038/s44271-026-00495-8)
Supplement: Supplementary file 3 — Description of Additional Supplementary Files [file 44271_2026_495_MOESM3_ESM.pdf]

Description of Additional Supplementary Files

File name- Supplementary Data 1

Description – Details of literature search

File name- Supplementary Data 2

Description - Variance distribution within- and between-person level
