# Supplementary material for: A comparative multi-method re-analysis of the longitudinal evidence for causal intergroup contact effects on attitudes
Source: Commun Psychol. 2026 Jul 15;4:107. doi: 10.1038/s44271-026-00495-8 (PMC13372666; doi:10.1038/s44271-026-00495-8)
Supplement: Supplementary file 1 — Transparent Peer Review file [file 44271_2026_495_MOESM1_ESM.pdf]

nature portfolio

Peer Review File

A Comparative Multi-Method Re-Analysis of the Longitudinal Evidence for Causal Intergroup Contact Effects on Attitudes

Corresponding Author: Dr Maria-Therese Friehs

This file contains all editorial decision letters in order by version, followed by all author rebuttals in order by version.

Version 0:

Decision Letter:

** Please ensure you delete the link to your author homepage in this e-mail if you wish to forward it to your coauthors **

Dear Professor Christ,

Thank you for your patience during the peer-review process. Your manuscript titled "What is the Longitudinal Evidence for Causal Intergroup Contact Effects? - A Comparative Multi-Method Re-Analysis of 21 Published Studies" has now been seen by 3 reviewers, and I include their comments at the end of this message. They find your work of interest but raised some important points. We are interested in the possibility of publishing your study in Communications Psychology, but would like to consider your responses to these concerns and assess a revised manuscript before we make a final decision on publication.

We therefore invite you to revise and resubmit your manuscript, along with a point-by-point response to the reviewers. Please highlight all changes in the manuscript text file.

Editorially, we consider it important that you address all methodological and conceptual concerns raised by the reviewers.

We are committed to providing a fair and constructive peer-review process. Please don't hesitate to contact us if you wish to discuss the revision in more detail.

I am attaching an Editorial Requests Table that details critical reporting requirements for the revised manuscript. Please attend to each item and ensure your manuscript is fully compliant. If your revised manuscript is not aligned with these requests on major issues, such as those concerning statistics, it may be returned to you for further revisions without re-review.

Please submit the following items:

- Revised manuscript
- Point-by-point response to the referees' comments
- Cover letter (as a separate document)
- Nature Research Reporting Summary
- Completed Editorial Request Table (attached).

via this link: Link Redacted .

** This url links to your confidential home page and associated information about manuscripts you may have submitted or be

reviewing for us. If you wish to forward this email to co-authors, please delete the link to your homepage first **

Additional guidance is available in our style and formatting guide Communications Psychology formatting guide.

We hope to receive your revised paper within 8 weeks; please let us know if you aren't able to submit it within this time so that we can discuss how best to proceed. If we don't hear from you, and the revision process takes significantly longer, we may close your file. In this event, we will still be happy to reconsider your paper at a later date, provided it still presents a significant contribution to the literature at that stage.

We would appreciate it if you could keep us informed about an estimated timescale for resubmission, to facilitate our planning.

We look forward to seeing the revised manuscript and thank you for the opportunity to review your work.

Best regards,

Jennifer Bellingtier

on behalf of
Hu Chuan-Peng, PhD
Editorial Board Member
Communications Psychology
orcid.org/0000-0002-7503-5131

REVIEWER EXPERTISE:
Reviewer #1 cross-lagged panel models, longitudinal data
Reviewer #2 cross-lagged panel models, data modeling
Reviewer #3 contact theory

REVIEWER REPORTS:

Reviewer #1 (Remarks to the Author):

Although I am not deeply familiar with the contact–attitude literature, I welcome the opportunity for multiple fields to engage in in-depth methodological reflection on the causal inference challenges raised by cross-lagged panel models (CLPM). From a methodological standpoint, I have several suggestions that I believe will strengthen the manuscript:

Sample size criterion for inclusion in the re-analysis
The decision to include only studies with N ≥ 100 participants appears arbitrary. Given that within-subject and between-subject statistical power can differ substantially depending on the number of waves, it would be more rigorous to conduct a priori power analyses to determine the minimum required sample size for different wave counts. This would allow the inclusion criteria to be grounded in statistical justification rather than a fixed threshold.

Measurement invariance
I did not find evidence that the authors examined measurement invariance for the variables, particularly for multi-item attitude measures in the re-analyzed studies. Even if the manuscript does not engage in latent mean comparisons, the interpretation of path coefficients in longitudinal structural equation models still requires at least metric invariance. Without this, differences in estimated paths may conflate changes in construct meaning with true change.

Theoretical assumptions of RI-CLPM
While I am not a domain expert on contact–attitude theory, the use of RI-CLPM entails two strong theoretical assumptions:
a. The focal relationships exist at the within-person level.
b. Time-invariant unobserved confounders are adequately captured at the between-person level (i.e., the between-subject effect is assumed not to vary over time).

For assumption (a), the introduction should explicitly raise this point and connect it to theory in the contact–attitude literature to justify why the within-person effect is the parameter of interest. This issue should also be revisited in the discussion to consider its implications for interpretation.

For assumption (b), the plausibility of the time-invariance assumption depends partly on the time scale of the study. It may be reasonable for short time intervals, but it becomes more problematic for studies spanning longer periods, as the proportion of trait versus state variance can change over time. In such cases, Trait–State–Occasion (TSO) models may provide a useful complementary framework for evaluating the stability of individual differences across the measurement period.

Overall, I appreciate the authors' efforts to systematically compare CLPM, FF-CLPM, and RI-CLPM in the context of

intergroup contact research. Addressing the above methodological points will further enhance the robustness and interpretability of the findings.

Reviewer #2 (Remarks to the Author):

What is the Longitudinal Evidence for Causal Intergroup Contact Effects? - A Comparative Multi-Method Re-Analysis of 21 Published Studies

Review

I think this is a neat, preregistered paper with a meaningful goal of re-analyzing previous results with modern approaches to causal inference with panel data. I like a lot of the manuscript. I will focus only on my points of criticism.

General
In general, I find the manuscript assumes too much knowledge on the part of the reader. While I'm quite familiar with the RI-CLPM, I can't say the same about the FF-CLPM. I think the paper could be stronger if the authors took time to really explain what makes these models equipped to make causal statements under weaker assumptions.

On that point, I don't care for the characterization of the RI-CLPM by the authors. They write, for example, "the CLPM considers unmeasured confounders only insufficiently" which is vague and doesn't properly frame the RI-CLPM. And page 28, lines 268-276 come close to conflating the separation of between- and within-variation with causal inference. To me, this is the main cautionary point of Rohrer & Murayama (2023) which the authors even cite.

Also related, I think the entire premise of the manuscript could be strengthened by really walking the reader through trying to make causal statements about intergroup contact and prejudice. What concrete real-world stable variables could confound the effect of intergroup contact on prejudice and vice versa? And how do the RI-CLPM and FF-CLPM address these confounders?

Methodological
Several studies the paper replicates involve samples of school-aged children and teenagers. If autoregressive and cross-lagged effects are constrained to be constant over time, then the RI-CLPM is an ongoing endogenous kind of autoregressive latent trajectory model (without the random slopes), see for example Ou et al. (2017). This type of model has built-in assumptions about the repeated measures under investigation, namely that they are at equilibrium (the "stationarity assumption"). For the current paper, that would mean individuals' attitudes towards outgroups have "settled" around some long-term average. I don't know if this is plausible for young people: probably, their attitudes are changing over time at least until they reach adulthood. Long story short: the RI-CLPM may return biased AR and CL effects in these situations. A quick way to check would be to see if the means, variances and (first-order) autocorrelations are roughly equal over the duration of the study, see Andersen (2022). Also, in simulations I've done, if the data haven't reached equilibrium, then the RI-CLPM displays poor fit.

I often read sentences like the one on page 28, lines 282-284: "This focus on within-person processes makes the cross-lagged effects of RI-CLPM difficult to compare to similar parameters in conventional CLPM and FF-CLPM from a substantial perspective." I think this oversells the RI-CLPM as something special when it can often be re-expressed as a more convoluted version of a traditional CLPM (just with unit or "random" effects). I think in most cases it's just the effect on Y of increasing X by one unit, either holding time-invariant confounders constant (RI-CLPM), or not (CLPM), see for example papers cited in last paragraph, as well as Lüdtke & Robitzsch (2022).

I'm having some trouble with the authors' criteria for adequate fit. RMSEA < .08 and SRMS < .1 is quite permissive. I would also like to know the chi-square its p-value. As it stands, the summary of model fit in table 2 is quite broad and doesn't tell me much.

Smaller points
I find this sentence muddles the premise: "However, given that these findings sit at the front of more than 70 years of research, which should not be discounted easily, in this work, we aim to investigate the extent to which evidence for longitudinal contact-attitude effects might be affected by different statistical models" (p. 8, lines 147-149). Either the prior research is robust and cumulative and deserves deference, or it is methodologically outdated and must be re-examined.

Maybe personal taste but Table 3 bugs me. The rows describing the type of contact ("Predictor: xxx") are awkward. They break up the visual flow, make it harder to scan. Maybe make two tables instead?

Referenced literature
Andersen, H. (2022). Equivalent Approaches to Dealing with Unobserved Heterogeneity in Cross-Lagged Panelm Models? Investigating the Benefits and Drawbacks of the Latent Curve Model with Structured Residuals and the Random Intercept Cross-Lagged Panel Model. Psychological Methods, 27(5), pp. 730-751. doi:10.1037/met0000285
Lüdtke, O., & Robitzsch, A. (2022). A Comparison of Different Approaches for Estimating Cross-Lagged Effects from a Causal Inference Perspective. Structural Equation Modeling: A Multidisciplinary Journal, 29(6), pp. 888-907. doi:10.1080/10705511.2022.2065278
Ou, L., Chow, S.-M., Ji, L., & Molenaar, P. (2017). (Re)evaluating the Implications of the Autoregressive Latent Trajectory

Model Through Likelihood Ratio Tests of Its Initial Conditions. Multivariate Behavioral Research, 52(2), pp. 178-199. doi:10.1080/00273171.2016.1259980
Rohrer, J., & Murayama, K. (2023). These Are Not the Effects You Are Looking For: Causality and the Within-/Between-Persons Distinction in Longitudinal Data Analysis. Advances in Methods and Practices in Psychological Science, 6(1), pp. 1-14. doi:10.1177/25152459221140842

Reviewer #3 (Remarks to the Author):

I was asked to review this article as the “the conceptual/theory expert” due to my past work on contact theory. My experience using cross-lagged panel models is somewhat limited. As a result, I will focus most of my review to how contact theory is discussed, leaving the technical details of your (re-)analyses for other reviewers to assess.

Overall, I think this work is important. As the authors point out, standard CLPMs are commonly used in intergroup contact research and if they produce biased estimates (and there are readily available alternatives) then that is a valuable insight for this area of research.

Your discussion of contact theory was to the point. It was accurate (to my knowledge) and efficient. However, I think that a more detailed discussion of contact theory may be useful to readers.

For those who don’t know much about contact theory, it could be useful just to discuss a few specific instances of contact reducing prejudice in the introduction – it’s important for readers to know contact is generally assumed to improve intergroup relations not only between groups that don’t know much about each other, but often groups with a long history of conflict band animosity between them.

It is also important to provide more context to previous research on negative intergroup contact. In your analyses (as shown in Table 3) you break the analyses into positive/unspecified contact and negative contact, which makes sense given previous research (e.g., Barlow et al., 2012). But negative contact is never discussed in the introduction of the paper. If someone is not well-versed in the contact literature, they may wonder why you made that methodological decision (as it is also not justified in the analysis plan). This paper would be strengthened by a deeper discussion of previous intergroup contact research in the introduction (including negative contact), an explicit justification for why you’re splitting contact effects the way you are in your analysis/results, and then also including a brief summary of what this distinction tells us in the discussion section.

In the discussion, you mention that these types of analyses may be useful in domains outside of intergroup contact. Do you have any insights/speculations as to why RI-CLPMs appear to outperform standard CLPMs in this context? Should we expect RI-CLPMs to improve model fit across any type of panel data, and therefore use RI-CLPMs by default in psychological research, or is there something about intergroup contact that makes those models well suited to it? You described your assumptions about intergroup contact effects in the discussion, but I wasn’t personally able to make the connection between that and why you think you found what you found.

Thanks for conducting this important work and I hope these comments are useful in improving this manuscript. Best of luck!

* TRANSPARENT PEER REVIEW: Communications Psychology uses a transparent peer review system. This means that we publish the editorial decision letters including Reviewers' comments to the authors and the author rebuttal letters online as a supplementary peer review file. However, on author request, confidential information and data can be removed from the published reviewer reports and rebuttal letters prior to publication. If your manuscript has been previously reviewed at another journal, those Reviewers' comments would not form part of the published peer review file.

** Visit Nature Research's author and referees' website at www.nature.com/authors for information about policies, services and author benefits**

Communications Psychology is committed to improving transparency in authorship. As part of our efforts in this direction, we are now requesting that all authors identified as ‘corresponding author’ create and link their Open Researcher and Contributor Identifier (ORCID) with their account on the Manuscript Tracking System prior to acceptance. ORCID helps the scientific community achieve unambiguous attribution of all scholarly contributions. You can create and link your ORCID from the home page of the Manuscript Tracking System by clicking on ‘Modify my Springer Nature account’ and following the instructions in the link below. Please also inform all co-authors that they can add their ORCIDs to their accounts and that they must do so prior to acceptance.
https://www.springernature.com/gp/researchers/orcid/orcid-for-nature-research

For more information please visit http://www.springernature.com/orcid

If you experience problems in linking your ORCID, please contact the Platform

Support Helpdesk.

Version 1:

Decision Letter:

** Please ensure you delete the link to your author homepage in this e-mail if you wish to forward it to your coauthors **

Dear Dr Friehs,

Your manuscript titled "Is there Longitudinal Evidence for Causal Intergroup Contact Effects? - A Comparative Multi-Method Re-Analysis of 21 Published Studies" has now been seen by our reviewers, whose comments appear below. In light of their advice I am delighted to say that we are happy, in principle, to publish a suitably revised version in Communications Psychology.

We therefore invite you to revise your paper one last time to address the remaining concerns of our reviewers and a list of editorial requests. At the same time we ask that you edit your manuscript to comply with our format requirements and to maximise the accessibility and therefore the impact of your work.

EDITORIAL REQUESTS:

Please review our specific editorial comments and requests regarding your manuscript in the attached "Editorial Requests Table". Please outline your response to each request in the right hand column. Please upload the completed table with your manuscript files as a Related Manuscript file.

If you have any questions or concerns about any of our requests, please do not hesitate to contact me.

SUBMISSION INFORMATION:

In order to accept your paper, we require the files listed here https://www.nature.com/documents/commsj-file-checklist.pdf .

OPEN ACCESS:

Communications Psychology is a fully open access journal. Articles are made freely accessible on publication. For further information about article processing charges, open access funding, and advice and support from Nature Research, please visit https://www.nature.com/commspsychol/open-access

At acceptance, you will be provided with instructions for completing the open access licence agreement on behalf of all authors. This grants us the necessary permissions to publish your paper. Additionally, you will be asked to declare that all required third party permissions have been obtained, and to provide billing information in order to pay the article-processing charge (APC).

* TRANSPARENT PEER REVIEW: Communications Psychology uses a transparent peer review system. On author request, confidential information and data can be removed from the published reviewer reports and rebuttal letters prior to publication. If you are concerned about the release of confidential data, please let us know specifically what information you would like to have removed. Please note that we cannot incorporate redactions for any other reasons.

* CODE AVAILABILITY: All Communications Psychology manuscripts must include a section titled "Code Availability" at the end of the methods section. We require that the custom analysis code supporting your conclusions is made available in a publicly accessible repository at this stage; please choose a repository that generates a digital object identifier (DOI) for the code; the link to the repository and the DOI must be included in the Code Availability statement. Publication as Supplementary Information will not suffice.

* DATA AVAILABILITY:
All Communications Psychology manuscripts must include a section titled "Data Availability" at the end of the Methods section. More information on this policy, is available in the Editorial Requests Table and at http://www.nature.com/authors/policies/data/data-availability-statements-data-citations.pdf.

Please use the following link to submit the above items:
Link Redacted
** This url links to your confidential home page and associated information about manuscripts you may have submitted or be reviewing for us. If you wish to forward this email to co-authors, please delete the link to your homepage first **

We hope to hear from you within two weeks; please let us know if you need more time.

Best regards,

Jennifer Bellingtier

Jennifer Bellingtier, PhD
Senior Editor
Communications Psychology

Hu Chuan-Peng, PhD
Editorial Board Member
Communications Psychology
orcid.org/0000-0002-7503-5131

REVIEWER EXPERTISE:
Reviewer #1 cross-lagged panel models, longitudinal data
Reviewer #3 contact theory

REVIEWERS' COMMENTS:

Reviewer #1 (Remarks to the Author):

The authors' revisions have satisfactorily addressed my concerns, and I have no further comments at this stage.

Reviewer #3 (Remarks to the Author):

Thanks for your thoughtful revisions and I appreciated you tagging the specific reviewer comments you were addressing within the document, that made it easy to follow (even with the tracked changes issues).

My concerns and recommendations that I outlined in my initial review have been satisfactorily addressed. Thanks for putting together an interesting and important paper.

** Visit Nature Research's author and referees' website at www.nature.com/authors for information about policies, services and author benefits**

Open Access This Peer Review File is licensed under a Creative Commons Attribution 4.0 International License, which permits use, sharing, adaptation, distribution and reproduction in any medium or format, as long as you give appropriate credit to the original author(s) and the source, provide a link to the Creative Commons license, and indicate if changes were made.
In cases where reviewers are anonymous, credit should be given to 'Anonymous Referee' and the source.
The images or other third party material in this Peer Review File are included in the article’s Creative Commons license, unless indicated otherwise in a credit line to the material. If material is not included in the article’s Creative Commons license and your intended use is not permitted by statutory regulation or exceeds the permitted use, you will need to obtain permission directly from the copyright holder.
To view a copy of this license, visit https://creativecommons.org/licenses/by/4.0/

Overview of responses to the feedback of the reviewers

Dear Dr. Bellingtier, dear anonymous reviewers,

We thank you very much for the opportunity to revise and resubmit our manuscript "*Is there longitudinal evidence for causal intergroup contact effects? – A comparative multi-method re-analysis of 21 published studies*". We express our sincere gratitude to the editor Dr. Bellingtier and the three anonymous reviewers, whose comments and suggestions on the initial version of the manuscript were invaluable and have assisted us in creating a stronger revised version of the manuscript. We believe the revised version now features more methodological rigor, accessibility to the reader, and a clearer storyline with more discussion and deliberation parts.

Please find below the relevant parts of all three reviewers' feedback plus a point-by-point explanation of how we handled the queries. We also refer you to specific passages of the track-changes version of the manuscript to demonstrate the implementation in the text. We apologize that the track-changes function in Microsoft Word provided unsatisfactory information about manuscript changes in the theory and discussion part (simply deleting and adding whole paragraphs). For easier orientation, the edited text in the track-changes version of the manuscript is commented to include references to the reviewers' feedback as outlined below.

Thank you very much for your effort in reviewing this manuscript.

Yours sincerely

The authors

Explanations of responses

Free-text response to a specific comment

"Quote from the revised manuscript"

"new text passages in the original text"

Reviewer # 1

R1.1: (…)

Sample size criterion for inclusion in the re-analysis
The decision to include only studies with N ≥ 100 participants appears arbitrary. Given that within-subject and between-subject statistical power can differ substantially depending on the number of waves, it would be more rigorous to conduct a priori power analyses to determine the minimum required sample size for different wave counts. This would allow the inclusion criteria to be grounded in statistical justification rather than a fixed threshold.

We thank the reviewer for this important observation and completely agree with their view on the relevance of statistical power. At the time of the initial pre-registration of the project in July 2021

(with an update in January 2022), there were scarce resources to determine power for complex structure equation models. Additionally, we needed to consider the state of the research field we re-analysed, which in many cases featured only medium-sizes samples. Therefore, we believed that N *= 100 would be the absolute minimum.*

However, following your advice, we have now conducted a posteriori *power analyses for all three analytical models featuring different sample sizes representative of our re-analysed dataset (100, 200, 300, 500, 800, 1,000, 2,000 participants), different numbers of measurement waves (3, 4, 5), and different effect sizes of the longitudinal contact-attitude effects (please see supplementary table 5). Overall, the results indicate that the power might be less than the general reference of 80% for samples with less than* N *= 2,000, which is about 75% of the re-analysed samples. We have added this information in the methods part (see p. 41-42 of the track-changes manuscript, lines 14-5) and in the discussion (see p. 56 of the track-changes manuscript, lines 7-14) of the manuscript.*

R1.2:

Measurement invariance

I did not find evidence that the authors examined measurement invariance for the variables, particularly for multi-item attitude measures in the re-analyzed studies. Even if the manuscript does not engage in latent mean comparisons, the interpretation of path coefficients in longitudinal structural equation models still requires at least metric invariance. Without this, differences in estimated paths may conflate changes in construct meaning with true change.

We thank the reviewer very much for pointing out this lack of information and fully acknowledge the value of testing measurement invariance. While we use observed mean values in the case of multi-item scales, metric measurement invariance over time is still a pre-requisite for such analyses. Consequently, we performed test of (partial) metric measurement invariance for all datasets featuring multi-item scales (under the condition that we had access to the raw items). The results of the measurement invariance assessment are reported in the individual R Markdown files documenting the full analyses and in supplementary table 3). (Partial) metric measurement invariance over time was achieved for all scales but one two-item scale of positive contact. We have added information concerning measurement invariance in the methods part (see p. 28 of the track-changes manuscript, lines 15-17) of the manuscript.

R1.3:

Theoretical assumptions of RI-CLPM

While I am not a domain expert on contact–attitude theory, the use of RI-CLPM entails two strong theoretical assumptions:

a. The focal relationships exist at the within-person level.

b. Time-invariant unobserved confounders are adequately captured at the between-person level (i.e., the between-subject effect is assumed not to vary over time).

For assumption (a), the introduction should explicitly raise this point and connect it to theory in the contact–attitude literature to justify why the within-person effect is the parameter of interest. This issue should also be revisited in the discussion to consider its implications for interpretation.

We thank the reviewer very much for these two essential observations which we had not addressed in the initial version of the manuscript. Indeed, from a theoretical perspective, intra-individual change in contact and attitudes (as opposed to stable between-person differences) are of interest for researchers, for which reason a number of contact intervention studies exist. We have added this information in the introduction (see p. 15 of the track-changes manuscript, lines 18-23) of the manuscript.

For assumption (b), the plausibility of the time-invariance assumption depends partly on the time scale of the study. It may be reasonable for short time intervals, but it becomes more problematic for studies spanning longer periods, as the proportion of trait versus state variance can change over time. In such cases, Trait–State–Occasion (TSO) models may provide a useful complementary framework for evaluating the stability of individual differences across the measurement period. (…)

We agree with the reviewer that the argument of increased causal inference in RI-CLPM relies strongly on the assumption that the stable between-person differences adequately capture time-invariant unobserved confounders. We know from some publications on intergroup contact that the stable between-person differences in contact and attitudes are predicted by demographic (e.g., age, gender), personality (e.g., agreeableness) and ideological (e.g., right-wing authoritarianism and social dominance orientation) variables (Friehs et al., 2024, DOI: 10.1177/19485506231153017; Shulman et al., 2024, DOI: 10.1177/19485506241284079). We are also aware of the current methodological discussion around illusory traits in the between-person components of the RI-CLPM. We have, however, not addressed this specific issue in the manuscript because the bias in causal inference that is introduced by time-varying unobserved variables affects all three models (i.e., CLPM, FF-CLPM, RI-CLPM) to the same degree and because the FF-CLPM and the within-person part of the RI-CLPM, which treat time-invariant unobserved confounders differently, but to a greater extent than the conventional CLPM, show similar results which diverge from the conventional CLPM results. We hope this response addresses your observation adequately.

Reviewer 2

R2.1: (…)

General
In general, I find the manuscript assumes too much knowledge on the part of the reader. While I'm quite familiar with the RI-CLPM, I can't say the same about the FF-CLPM. I think the paper could be stronger if the authors took time to really explain what makes these models equipped to make causal statements under weaker assumptions.

We thank the reviewer for this important observation and fully agree that the introduction of the statistical models was quite short in the initial version of the manuscript. We have now added substantial information about the statistical models, including a comprehensive illustration from contact research (see also points R2.2 and R2.3), in the introduction (see p. 14, line 21 to p. 16, line 14 of the track-changes manuscript, Theory section).

R2.2:

On that point, I don't care for the characterization of the RI-CLPM by the authors. They write, for example, "the CLPM considers unmeasured confounders only insufficiently" which is vague and doesn't properly frame the RI-CLPM. And page 28, lines 268-276 come close to conflating the separation of between- and within-variation with causal inference. To me, this is the main cautionary point of Rohrer & Murayama (2023) which the authors even cite.

Thank you very much for pointing out this vagueness. We have now explained in more detail how the RI-CLPM addresses the issue of unobserved time-invariant confounders and made an effort to emphasize that the within-between variance differentiation in the RI-CLPM does not directly relate to causal inference (see p. 15, line 10 to p. 16, line 14 of the track-changes manuscript, Theory part, as well as p. 40 of the track-changes manuscript, lines 15-23).

R2.3:

Also related, I think the entire premise of the manuscript could be strengthened by really walking the reader through trying to make causal statements about intergroup contact and prejudice. What concrete real-world stable variables could confound the effect of intergroup contact on prejudice and vice versa? And how do the RI-CLPM and FF-CLPM address these confounders?

We thank the reviewer for this excellent suggestion. As mentioned in point R2.1, we have added such information including an illustrative example in the introduction:

"Inter alia, both contact and attitudes are related to personality[36,37] and intergroup ideologies[38] – for instance, an individual high in right-wing authoritarianism (RWA) tends to have unfavourable attitudes towards outgroups[39,40] and also to have low levels of intergroup contact[41]. As RWA is related both to contact and attitudes, it potentially confounds the longitudinal contact-attitude effects because a potentially observed link between a person having low contact$_{T1}$ and unfavourable outgroup attitudes$_{T2}$ may be explained by that person being persistently high in RWA. Problematically, it has long been shown that the CLPM produces biased causal estimates in the presence of unmeasured confounders at all measurement waves. That is, if the RWA level of participants affects their intergroup contact and outgroup attitude levels at all measurement waves, the CLPM will most likely produce biased estimates of the longitudinal contact-attitude

effect. Consequently, the causal evidence provided by CLPM is questionable[21,42–46] (for a technical explanation of bias in causal estimates in CLPM, we recommend Murayama & Gfrörer, 2024)."
(p. 14 of the track-changes manuscript, lines 4-16, Theory section)

"Using the example above, if RWA serves as an unobserved time-invariant confounder affecting intergroup contact and outgroup attitudes levels at all timepoints, higher-order stability and lagged effects reflect the accumulated history of intergroup contact experiences and attitude formation (which has already been exposed to the confounding effect of RWA)."
(p. 15 of t he track-changes manuscript, lines 3-6, Theory section)

"Using the example above, in an RI-CLPM, the time-invariant confounding influence of RWA is modelled as part of the stable between-person differences in intergroup contact and outgroup attitudes (or their covariation), which are modelled statistically independent from the estimation of the longitudinal contact-attitude effects."
(p. 16 of the track-changes manuscript, lines 6-9, Theory section

R2.4:

Methodological
Several studies the paper replicates involve samples of school-aged children and teenagers. If autoregressive and cross-lagged effects are constrained to be constant over time, then the RI-CLPM is an ongoing endogenous kind of autoregressive latent trajectory model (without the random slopes), see for example Ou et al. (2017). This type of model has built-in assumptions about the repeated measures under investigation, namely that they are at equilibrium (the "stationarity assumption"). For the current paper, that would mean individuals' attitudes towards outgroups have "settled" around some long-term average. I don't know if this is plausible for young people: probably, their attitudes are changing over time at least until they reach adulthood. Long story short: the RI-CLPM may return biased AR and CL effects in these situations. A quick way to check would be to see if the means, variances and (first-order) autocorrelations are roughly equal over the duration of the study, see Andersen (2022). Also, in simulations I've done, if the data haven't reached equilibrium, then the RI-CLPM displays poor fit.

We thank the reviewer for this thought and agree with the mentioned concern. However, for all analyses, we tested for stationarity by comparing a model with similarity restrictions for all stability and cross-lagged effects to a freely estimated model with varying stability and cross-lagged coefficients. If the robust maximum likelihood (MLR)-corrected chi-square difference test was non-significant, we assumed stationarity (as introducing similarity constraints did not significantly worsen model fit). However, in case of a significant chi-square difference test, we used the freely estimated models for interpretation. Please see supplementary table 4 for full results. Additionally, the mini meta-analysis now only contains freely estimated models, as stationarity could not be assumed for many models. We have not explicitly investigated whether children/teenager or any other population or context were more or less likely to produce stationary results.

R2.5:

I often read sentences like the one on page 28, lines 282-284: "This focus on within-person processes makes the cross-lagged effects of RI-CLPM difficult to compare to similar parameters in conventional CLPM and FF-CLPM from a substantial perspective." I think this oversells the RI-CLPM as something special when it can often be re-expressed as a more convoluted version of a traditional CLPM (just

with unit or “random” effects). I think in most cases it’s just the effect on Y of increasing X by one unit, either holding time-invariant confounders constant (RI-CLPM), or not (CLPM), see for example papers cited in last paragraph, as well as Lüdtke & Robitzsch (2022).

We thank the reviewer for this observation and have deleted such information from the revised version of the manuscript.

R2.6:

I’m having some trouble with the authors’ criteria for adequate fit. RMSEA < .08 and SRMS < .1 is quite permissive. I would also like to know the chi-square its p-value. As it stands, the summary of model fit in table 2 is quite broad and doesn’t tell me much.

We thank the reviewer for this observation. We have now provided full model fit information including chi-square tests and accompanying p-values *in supplementary table 4.*
We are aware of a number of different recommendations for model fit cut-off criteria, including dynamic ones which are adjusted specifically to the analyses at hand (McNeish & Wolf, 2023, DOI: 10.1037/met0000425*). Thus, a variety of cut-off criteria was possible and plausible, and we decided on this specific set of criteria, which was broadly received in psychological literature and has been cited more than 4,000 times according to APA PsycNet. Although more restrictive cut-off recommendations exist, we believe that our selection is plausible. What is more, our re-analysis shows that model fit of the CLPM is oftentimes insufficient, which is even more cause for concern as we use rather lenient model fit criteria. The RI-CLPM shows substantially improved and mostly acceptable model-fit, which in most cases would also hold when using stricter criteria (see supplementary table 4, where we list the full model fit information for all analysed models). For the FF-CLPM, no model fit evaluation is possible as the model is just-identified by nature.*

R2.7:

Smaller points
I find this sentence muddles the premise: “However, given that these findings sit at the front of more than 70 years of research, which should not be discounted easily, in this work, we aim to investigate the extent to which evidence for longitudinal contact-attitude effects might be affected by different statistical models” (p. 8, lines 147-149). Either the prior research is robust and cumulative and deserves deference, or it is methodologically outdated and must be re-examined.

We thank the reviewer for this observation and have revised the statement, which now reads:

*“*However, these findings are based on a small number of longitudinal data which are not representative for the complete field of longitudinal intergroup contact research and which do not necessarily align with previously published works. Additionally, these findings are inconsistent with the overall results of more than 70 years of contact research (including randomised experiments)”
(p. 17 of the track-changes manuscript, lines 4-8, Theory section)

R2.8:

Maybe personal taste but Table 3 bugs me. The rows describing the type of contact ("Predictor: xxx") are awkward. They break up the visual flow, make it harder to scan. Maybe make two tables instead? (…)

We thank the reviewer for this observation and have now separated the table (distinct tables for positive/non-valenced and negative contact) and reduced its contents (removing information about stationary models, as this assumption did not hold for all tested models).

Reviewer 3

R3.1: (…)

For those who don't know much about contact theory, it could be useful just to discuss a few specific instances of contact reducing prejudice in the introduction – it's important for readers to know contact is generally assumed to improve intergroup relations not only between groups that don't know much about each other, but often groups with a long history of conflict band animosity between them.

We thank the reviewer for this thoughtful observation. In the current version of the manuscript, we have extended the introduction of contact theory to include contact effects in peaceful and (intractable) conflict-ridden contexts, the differential effects of positive and negative contact (see also R3.2), and cross-group friendships as an especially potent form of positive contact. We wrote:

"Contact research has historically focused on the effects of non-valenced or (implicitly or explicitly) positive contact, with intergroup friendships[3,4] as most optimal instances of intergroup contact which show high impact on outgroup attitudes. Intergroup contact has demonstrated its effectiveness not only for unknown outgroups in peaceful societies, but also in instances of persisting segregation, severe intergroup conflict and animosity, for instance in post-conflict settings[5–7]."
(p. 11 of the track-changes manuscript, lines 5-13, Theory section)

R3.2:

It is also important to provide more context to previous research on negative intergroup contact. In your analyses (as shown in Table 3) you break the analyses into positive/unspecified contact and negative contact, which makes sense given previous research (e.g., Barlow et al., 2012). But negative contact is never discussed in the introduction of the paper. If someone is not well-versed in the contact literature, they may wonder why you made that methodological decision (as it is also not justified in the analysis plan). This paper would be strengthened by a deeper discussion of previous intergroup contact research in the introduction (including negative contact), an explicit justification for why you're splitting contact effects the way you are in your analysis/results, and then also including a brief summary of what this distinction tells us in the discussion section.

We thank the reviewer for this important comment. As mentioned in our response to R3.1, we have added such explanations in the theory section. Additionally, the methods, results and discussion sections now include references to the distinction of positive and negative contact. Specifically,we wrote:

"Importantly, in contrast to the positive impact of non-valenced or positive contact, negative intergroup contact[8,9] has been identified as a distinct phenomenon in contact research affecting attitudes especially strongly and negatively."
(p. 11 of the track-changes manuscript, lines 10-13, Theory part)

"In the meta-analysis, we treated non-valenced contact, positive intergroup contact and cross-group friendships separately from negative intergroup contact, as the processes are quite distinct and affect outgroup attitudes in opposing directions."
(p. 41 of the track-changes manuscript, lines 11-13, Methods part)

". In RI-CLPM, the cross-sectional correlations of the random intercepts (i.e., between-person variance) of contact and attitudes showed significant effects in line with the predictions of contact

theory for both positive and negative contact. The sizes of the effects did not vary substantially between positive and negative contact."
(p. 44 of the track-changes manuscript, lines 16-20, Results section)

"These findings were observed consistently for non-valenced or positive and negative contact experiences."
(p. 53 of the track-changes manuscript, lines 19-20, Discussion section)

R3.3:

In the discussion, you mention that these types of analyses may be useful in domains outside of intergroup contact. Do you have any insights/speculations as to why RI-CLPMs appear to outperform standard CLPMs in this context? Should we expect RI-CLPMs to improve model fit across any type of panel data, and therefore use RI-CLPMs by default in psychological research, or is there something about intergroup contact that makes those models well suited to it? You described your assumptions about intergroup contact effects in the discussion, but I wasn't personally able to make the connection between that and why you think you found what you found. (…)

Thank you very much for this question, which we would like to answer directly in this response and which was indirectly addressed in the manuscript. Hamaker et al. (2015, DOI: 10.1037/a0038889) argue that CLPM and RI-CLPM are directly nested, as an RI-CLPM could be transferred into a CLPM by setting the variances and covariance of the random-intercepts/between-person effects to zero. Thus, as reviewer 2 put it, the RI-CLPM is a conventional CLPM with a unit effect/mean centering. Across different fields of psychology science, publications that have directly compared CLPM and RI-CLPM have found that the RI-CLPM shows usually substantially improved model fit (e.g., …). Thus, we believe that this effect is not specific to contact research. However, we argue that such improved model fit should not lead to the interpretation that the RI-CLPM should be used by default. Analytical models should rather be carefully evaluated and chosen so that their statistical estimand is well-suited for the specific research questions and study designs (for a more elaborated argument, see Rohrer & Murayama, 2023, DOI: 10.1177/25152459221140842; and Hamaker, 2023, DOI: 10.1037/met0000600). We have also emphasized this recommendation in the manuscript:

"This is oftentimes the case, also in applications outside contact research, because the CLPM can be considered a more restrictive version of the RI-CLPM in which the between-person factors and their covariance have been restricted to be zero[44], which might impede model-fit. Still, we wish to emphasize that in empirical research, there are a number of criteria for the selection of a specific analytical model besides model-fit, including theoretical considerations about the effect under investigation[49]. Thus, we do not recommend the usage of the RI-CLPM as a default based solely on model-fit."
(p. 43 of the track-changes manuscript, lines 10-16, Results section)
